# In Situ Mapping of Phase Evolutions in Rapidly Heated Zr‐Based Bulk Metallic Glass with Oxygen Impurities

**DOI:** 10.1002/advs.202307856

**Published:** 2024-02-28

**Authors:** Mattias Tidefelt, Julia Löfstrand, Inga K. Goetz, Olivier Donzel‐Gargand, Anders Ericsson, Xiaoliang Han, Petra E. Jönsson, Martin Sahlberg, Ivan Kaban, Martin Fisk

**Affiliations:** ^1^ Department of Materials Science and Applied Mathematics Malmö University Nordenskiöldsgatan 1 Malmö SE‐21119 Sweden; ^2^ Division of Materials Physics Department of Physics and Astronomy Uppsala University Box 530 Uppsala SE‐75121 Sweden; ^3^ Division of Solar Cell Technology Angström Solar Centre Department of Materials Science and Engineering Uppsala University Uppsala 75121 Sweden; ^4^ Division of Solid Mechanics Lund University P.O. Box 118 Lund SE‐221 00 Sweden; ^5^ Leibniz Institute for Solid State and Materials Research Helmholtzstr. 20 01069 Dresden Germany; ^6^ Department of Chemistry ‐ Angström Laboratory Uppsala University Box 538 Uppsala SE‐751 21 Sweden

**Keywords:** additive manufacturing, AMLOY‐ZR01, classical nucleation and growth theory, small‐angle X‐ray scattering, wide‐angle X‐ray scattering, transmission electron microscopy

## Abstract

Metallic glasses exhibit unique mechanical properties. For metallic glass composites (MGC), composed of dispersed nanocrystalline phases in an amorphous matrix, these properties can be enhanced or deteriorated depending on the volume fraction and size distribution of the crystalline phases. Understanding the evolution of crystalline phases during devitrification of bulk metallic glasses upon heating is key to realizing the production of these composites. Here, results are presented from a combination of in situ small‐ and wide‐angle X‐ray scattering (SAXS and WAXS) measurements during heating of Zr‐based metallic glass samples at rates ranging from 10^2^ to 10^4^ Ks^−1^ with a time resolution of 4ms. By combining a detailed analysis of scattering experiments with numerical simulations, for the first time, it is shown how the amount of oxygen impurities in the samples influences the early stages of devitrification and changes the dominant nucleation mechanism from homogeneous to heterogeneous. During melting, the oxygen rich phase becomes the dominant crystalline phase whereas the main phases dissolve. The approach used in this study is well suited for investigation of rapid phase evolution during devitrification, which is important for the development of MGC.

## Graphical abstract

Oxygen impurities impact on phase‐transformations during rapid heating of Zr‐based metallic glass Zr_59.3_Cu_28.8_Al_10.4_Nb_1.5_ is thoroughly investigated using a multi‐technique approach. During devitrification, the extracted phase evolutions reveal that the phase fraction hierarchy correlates with the oxygen impurity concentration. Numerical simulations with a heterogeneous nucleation mode capture the experimental observations. During melting, the oxygen‐rich phase becomes the dominant phase.

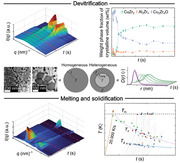

## 1 Introduction

The inherently excellent properties and promising applications of metallic glasses^[^ ^1^, ^2^, ^3^ ^]^ have motivated intensive research on the manufacturing of bulk metallic glasses (BMGs). The main challenge with application of BMGs is the difficulty in producing 3D parts of desirable shapes and sizes. Suction casting, commonly used for fabrication of BMG, can only provide parts with limited geometry (rods and plates), but can be further shaped with superplastic forming as these alloys exhibit a large super‐cooled liquid region with low viscosity.^[^ ^1^, ^4^ ^]^ However, for realizing large‐scale BMG objects without limitation in size and shape, additive manufacturing (AM) methods, in particular the laser powder‐bed fusion (LPBF) techniques,^[^ ^5^, ^6^, ^7^, ^8^ ^]^ are very promising and versatile since the elevated local heating and cooling rates allow exceeding the dimensions and geometries achievable by traditional methods.^[^ ^9^, ^10^ ^]^ One drawback of AM is that oxygen contamination is inevitable.^[^ ^11^ ^]^ Because of the high surface to volume ratio of the powder feedstock material, in combination with difficulties of controlling the atmosphere during the processing, produced parts exhibit much higher concentrations of oxygen impurities compared to cast material. In many glass‐forming systems, the presence of oxygen inhibits amorphization or accelerates the devitrification. For example, Zr‐based systems have been shown to be very sensitive to this contamination.^[^ ^1^, ^11^, ^12^, ^13^, ^14^ ^]^

Partial crystallization of BMG can enhance, or deteriorate, mechanical properties. Plastic flow is generally limited in BMGs as highly localized shear bands are formed during deformation, leading to catastrophic failure from heterogeneous deformation.^[^ ^1^ ^]^ By reinforcing the amorphous matrix with controlled volumes of nano‐sized ductile crystalline precipitates, creating metallic glass composites (MGC), this drawback can effectively be eliminated.^[^ ^15^, ^16^, ^17^, ^18^ ^]^ The precipitates can impede local shear band propagation or change its direction, resulting in a more uniform spatial distribution of shear bands, and thus in an increased ductility of the material. On the other hand, precipitates may exhibit a higher hardness than the amorphous matrix, resulting in a negative impact on the ductility of the alloy by promoting crack formation rather than shear band distribution, which is typically the case for oxygen‐rich phases.^[^ ^11^, ^19^ ^]^ For commercial production of Zr‐based BMG parts with reproducible properties detailed knowledge of how oxygen impurities impact the phase‐transformations in the alloys is essential. Furthermore, methods for mapping distributions of nano‐sized particles within an amorphous matrix are needed to understand and predict a glass forming system's capability of forming a composite structure.

The temperature history during AM processing within the different layers varies.^[^ ^20^ ^]^ Hence, to fully understand the phase evolution during processing, it is important to study the kinetics of individual phase‐transformations at different heating rates. In the Zr‐based Zr_59.3_Cu_28.8_Al_10.4_Nb_1.5_ alloy, known as AMLOY‐ZR01^[^ ^21^ ^]^ (formerly AMZ4) the oxygen‐induced phases Cu_2_Zr_4_O ($Fd\bar{3}m$).^[^ ^6^, ^7^, ^8^ ^]^ and α‐Zr(O) (*P*6_3_/*mmc*)^[^ ^7^ ^]^ have been reported to form during processing. Other phases formed upon heating are CuZr_2_ (*I*4/*mmm*),^[^ ^5^, ^6^, ^11^, ^22^ ^]^ Al_7_Cu_16_Zr_6_ ($Fm\bar{3}m$),^[^ ^5^ ^]^ Al_3_Zr_4_ (*P*6/*mmm*),^[^ ^6^, ^11^, ^22^ ^]^ and Al_2_Zr_3_.^[^ ^6^ ^]^ The $Fd\bar{3}m$ structure with (Cu,Ni)_2_Zr_4_O stoichiometry has also been reported to form in several other Zr‐based glass‐forming alloys, containing Cu and/or Ni.^[^ ^19^, ^23^, ^24^ ^]^ This oxygen induced structure has been suggested to induce a two step transformation sequence where the oxygen rich structure forms prior to equilibrium structures, promoting heterogeneous nucleation.^[^ ^13^, ^23^, ^25^ ^]^ In addition, in AMLOY‐ZR01 it has been shown that the thermal stability of the amorphous phase is severely reduced if the oxygen contamination reach concentrations of ≈1at.%.^[^ ^5^, ^7^, ^25^ ^]^ The temperature‐dependent devitrification kinetics have been mapped using flash differential scanning calorimetry (FDSC).^[^ ^25^, ^26^ ^]^ Although this technique allows determination of key temperatures and times at high heating rates, no information of the underlying phase evolution can be extracted.

Recently, Orava et al.^[^ ^27^ ^]^ performed in situ wide‐angle X‐ray scattering (WAXS) measurements during flash‐annealing of Zr_47.5_Cu_47.5_Al_5_ metallic glass using a Joule heating setup, capturing rapid phase‐transformations. To fully describe the crystal growth, it is also valuable to understand the crystallization process. One example of this is Lou et al., who successfully obtained details of the distribution parameters during clustering and crystal growth in a Ce_65_Al_10_Co_25_ metallic glass using small‐angle X‐ray scattering (SAXS).^[^ ^28^ ^]^ A combination of these two scattering techniques (SAXS/WAXS) was applied to study phase‐transformations in a Zr_46_Cu_46_Al_8_ metallic glass in situ during low‐temperature isothermal annealing by Wu et al.^[^ ^29^ ^]^ Supported by Kolmogorov‐Johnson‐Mehl‐Avrami (KJMA) modeling, it was shown that the process is dominated by homogeneous nucleation and diffusion‐controlled growth. With the other well‐known continuum approach of modeling, classical nucleation and growth theory (CNGT), Ericsson et al.^[^ ^30^ ^]^ produced simulation results decoupled from data fits that showed good agreement with experimentally obtained continuous heating transformation (CHT) diagrams by using a thermodynamic description of the Zr‐Cu‐Al system to mimic AMLOY‐ZR01. However, no consideration of phase fractions was taken, which is key to properly predict and understand a systems kinetic transformation behavior.

To obtain detailed information about the phase formation, the phase fractions, and the phase specific crystalline size distributions, we used a combination of in situ WAXS and SAXS techniques with a time resolution of 4 ms, ex situ microstructural analysis with transmission electron microscopy (TEM), and numerical simulations based on CNGT, to investigate the structural evolution in metallic glass samples based on the composition of AMLOY‐ZR01 with different concentrations of oxygen impurities. The samples were exposed to Joule heating at rates ranging from 10^2^ to 10^4^ Ks^−1^ (the samples with the highest heating rates had a different cross section compared to those with the lower heating rates) during which we probed the devitrification, following the phase evolution of the identified phases, their dissolution by further heating, and finally crystallization upon quenching. By changing the heating rate, it was possiblie to mimic the temperature histories at different spatial distances from the melt pool during the AM process.^[^ ^20^ ^]^ With numerical simulations, we present phase‐specific particle size distributions and demonstrate how a heterogeneous nucleation event impacts the nucleation rate and hence alters the crystalline phase fractions, which explains our experimental findings. The devitrified end product was further analyzed with TEM, including energy‐dispersive X‐ray spectroscopy (EDS). In summary, combining simultaneous WAXS and SAXS measurements during flash‐annealing with numerical devitrification modeling yields great insight into rapid temperature‐dependent phase evolution in AMLOY‐ZR01 with different oxygen concentrations, which is important for the development of bulk metallic glasses and MGC.

## 2 Results

### 2.1 The Devitrification Process

Several BMG sample pieces were flash‐annealed by passing constant currents in the range of 10–25 A through the material using a setup constructed by IFW Dresden.^[^ ^31^ ^]^ Assuming a linear heating rate from room temperature to the onset time and temperature of devitrification (*t*_*x*1_, *T*_*x*1_) resulted in an approximated linear heating rate, Φ_*lin*_, range of 10^2^–10^3^ Ks^−1^ (Φ_*lin*_ for all the measurements are found in Experimental data analysis, Supporting Information). During the synchrotron experiments, the resistivity of each sample was monitored simultaneously with the surface temperature at the longitudinal center of the sample. SAXS and WAXS patterns were taken every 4ms. The samples were synthesized in four series that contained impurity concentrations of oxygen in the range of 1.3–4.3at.%. For readability the sample pieces will henceforward be referred to as sample (i)–(iv), implying that different pieces from the series are used in the separate in‐situ measurements, where the heating process was varied by passing different currents through the samples. The oxygen content in the samples was analyzed with time‐of‐flight elastic recoil detection analysis (ToF‐ERDA).

**Figure 1:**
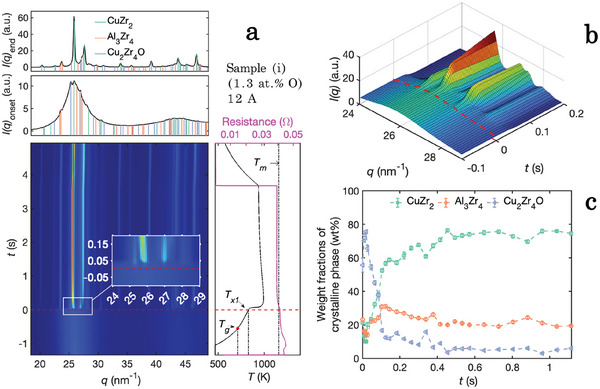
Structural phase ordering in sample (i) containing 1.3at.% O during flash‐annealing with a current of 12A resulting in an approximated linear heating rate of Φ_*lin*_ = 400Ks^−1^. Top (a): Diffraction pattern at the end of the measurements and at the onset of devitrification, *t*_*x*1_. Bottom (a): Integrated WAXS patterns from the flash‐anneal measurements. The inset shows a close up of the onset of devitrification. Right bottom part of (a): Time dependent temperature curves (black) and resistivity curves (pink). A sharp increase in temperature is observed at the exothermic nucleation event. The melting temperature *T*_*m*_, the onset of devitrification, *T*_*x*1_, and the glass transition temperature, *T*_*g*_, are marked with dashed black lines. The onset time of devitrification, *t*_*x*1_, is marked with a dashed red line. The glass transition temperature (*t*_*g*_, *T*_*g*_) is extracted by evaluating the change of the temperature gradient at temperatures lower than *T*_*x*1_. b) A 3D representation of the XRD intensities marked by the rectangle in panel (a). c) Weight phase fraction of the crystalline volume obtained by Rietveld refinements of the WAXS patterns.

**Figure 2:**
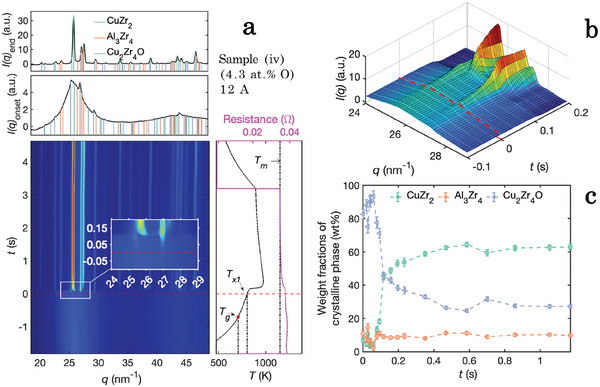
Structural phase ordering in sample (iv) containing 4.3at.% O during flash‐annealing with a current of 12A resulting in an approximated linear heating rate of Φ_*lin*_ = 280Ks^−1^. Top (a): Diffraction pattern at the end of the measurements and at the onset of devitrification, *t*_*x*1_. Bottom (a): Integrated WAXS patterns from the flash‐anneal measurements. The inset shows a close up of the onset of devitrification. Right bottom part of (a): Time dependent temperature curves (black) and resistivity curves (pink). A sharp increase in temperature is observed at the exothermic nucleation event. The melting temperature *T*_*m*_, the onset of devitrification, *T*_1_, and the glass transition temperature, *T*_*g*_, are marked with dashed black lines. The onset time of devitrification, *t*_*x*1_, is marked with a dashed red line. The glass transition temperature (*t*_*g*_, *T*_*g*_) is extracted by evaluating the change of the temperature gradient at temperatures lower than *T*_*x*1_. b) A 3D representation of the XRD intensities marked by the rectangle in panel (a). c) Weight phase fraction of the crystalline volume obtained by Rietveld refinements of the WAXS patterns.

In **Figures** **1** and **2**, WAXS results obtained from in situ flash‐annealing of the samples containing the lowest and highest amount of oxygen (sample (i) and sample (iv)) are presented. With a constant current of 12A and an annealing time of 5 s, each sample was heated to a temperature that caused devitrification, but was below the melting temperature. The approximated linear heating rates were calculated to be 400 and 280 Ks^−1^, respectively. With Rietveld refinement of the WAXS data the CuZr_2_ phase, Al_3_Zr_4_ phase, and Cu_2_Zr_4_O phase were identified. The onset time and temperature of devitrification that are marked in Figures 1 and 2 were extracted from the WAXS diffraction patterns in each sequence where the crystalline peaks first started to increase in relative intensity, indicating that the crystalline phases were growing. The weight fraction of the ordered phases calculated from refinements using the Rietveld method is presented in Figure 1c for sample (i) and Figure 2c sample (iv), respectively. Comparing the first 50ms after the onset of devitrification for sample (i) with sample (iv), Bragg reflection peaks that are solely attributed to the Cu_2_Zr_4_O phase are observed in sample (iv) (see Figure 2b). This is in contrast to sample (i), where reflections from all detected phases are observed simultaneously at *t*_*x*1_ = 0s (see Figure 1b). Figures 1c and 2c show that after just 100 ms of devitrification, the CuZr_2_ phase dominates the weight fraction of the crystalline volume, regardless of the oxygen content. In sample (i), the Al_3_Zr_4_ phase is the second largest contributor to the crystalline mass, but is altered to be the Cu_2_Zr_4_O phase in sample (iv). During the first 400ms of devitrification, the relative phase fractions are evolving, but after this point they stabilize for both samples.

Initial Bragg reflections from Cu_2_Zr_4_O are observed in some cast sample pieces, indicating that they were not fully amorphous in the as‐cast state. The correlation between the Cu_2_Zr_4_O and the phase formation sequence during the devitrification onset stays the same however, independent of initial crystallinity. A threshold oxygen concentration for the earlier formation of Cu_2_Zr_4_O seems to lie between sample (ii) and (iii) at around 2at.% oxygen. Therefore, we can conclude that the amount of oxygen changes the initial sequence of the onset of crystallization from all phases being formed simultaneously for oxygen concentrations (<2at.%), to having the Cu_2_Zr_4_O phase formed primary to the other phases at oxygen concentrations >2at.%. A representative selection of 3D visualizations from the WAXS measurements in the vicinity of *t*_*x*1_ for sample (i)‐(iv) can be found in the Experimental data analysis, Phase ordering sequence at the onset of devitrification (Supporting Information).

**Figure 3:**
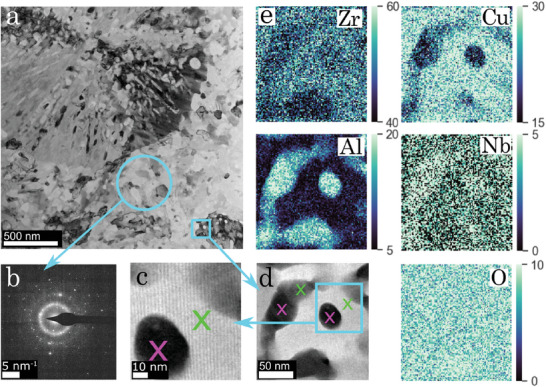
Ex situ TEM analysis showing the microstructural end product of the devitrified sample (i) containing 1.3at.% after flash‐annealing with a current of 12A. a) BF TEM micrograph of the final microstructure. b) SAED pattern of the selected area showing that the measured region is polycrystalline but also has a significant amount of amorphous phase as indicated by the broad halos. d) HAADF STEM micrograph representing a magnification of selected part of (a) where the crosses represent the places from which the CuZr_2_ (green) and Al_3_Zr_4_ (magenta) phases were identified with NBD. c) Magnification of selected part of (d) showing the long‐range order of the identified CuZr_2_ phase. e) EDS of the constituent elements from the region in (d), the color bars represent relative atomic concentration.

Micrographs from TEM (including bright field (BF), high angle annular dark field (HAADF) scanning TEM (STEM), selected area electron diffraction (SAED), and EDS) of sample (i) flash‐annealed with a constant current of 12A are presented in **Figure** **3**. The analysis was done close to the center of the sample piece, and the SAED clearly shows an amorphous remaining phase characterized by diffuse broad halos, mixed with the discrete diffraction spots from the crystalline phases (Figure 3b). The micrograph presented in Figure 3a shows variations in microstructure, suggesting a distribution of crystalline growth. In Figure 3d, crosses mark the regions where the CuZr_2_ phase (green) and Al_3_Zr_4_ phase (magenta) could be identified with nano‐beam diffraction (NBD). The region surrounding the rightmost crosses is displayed in Figure 3c where the crystalline long‐range order in CuZr_2_ is clearly visible.

With the NBD, a specific depth of the lamella can be probed whereas the EDS gives the average concentration for the whole region probed in transmission when performing the concentration mapping. The EDS results (Figure 3e) depict that the bright contrast region around the rightmost green cross is depleted in Al but rich in Cu, whereas the left green cross is located in a region with higher concentrations of Al. The elemental concentrations in this region can be explained by overlapping phases in the analyzed lamella, giving an intermediate Al and Cu concentration compared to the dark and bright regions. However, this cannot be conclusively distinguished from contrast variations stemming from different intermixing between Al and Cu. The dark contrast regions around the magenta crosses (Figure 3c) are rich in Al and contain lower concentrations of Cu compared to the light regions. Based on these findings, we conclude that the Cu‐rich phase (Cu,Al)Zr_2_ and Al‐rich phase (Cu,Al)_3_Zr_4_ are formed.^[^ ^32^ ^]^ The Nb shows slightly higher concentrations in the Al‐rich regions, and the oxygen concentration is evenly distributed throughout the analyzed region. The substitutional intermixing of atoms indicated in these results could unfortunately not be included to improve the structural analysis and refinements however, since it was not possible to distinguish this effect from other influences that can cause peak broadening and shifting, such as temperature‐related displacement of atoms and strain fields around the precipitates.

In sample (iv), distinct spherical/hexagonal particles were found, which were not observed in the probed region of sample (i). These particles could be identified as the Cu_2_Zr_4_O phase with SAED. HAADF STEM micrographs of the devitrified end product of sample (i) and (iv) further suggest that the dark contrast regions, which are rich in Al, decrease in size when the oxygen concentration is elevated. The TEM micrographs used to identify the phases in the samples (i) and (iv) can be found together with additional HAADF STEM and EDS micrographs of the microstructures in Experimental data analysis, Microstructural analysis with TEM (Supporting Information).

### 2.2 Thermally Driven Clustering and Growth of Precipitates During Devitrification

**Figure 4:**
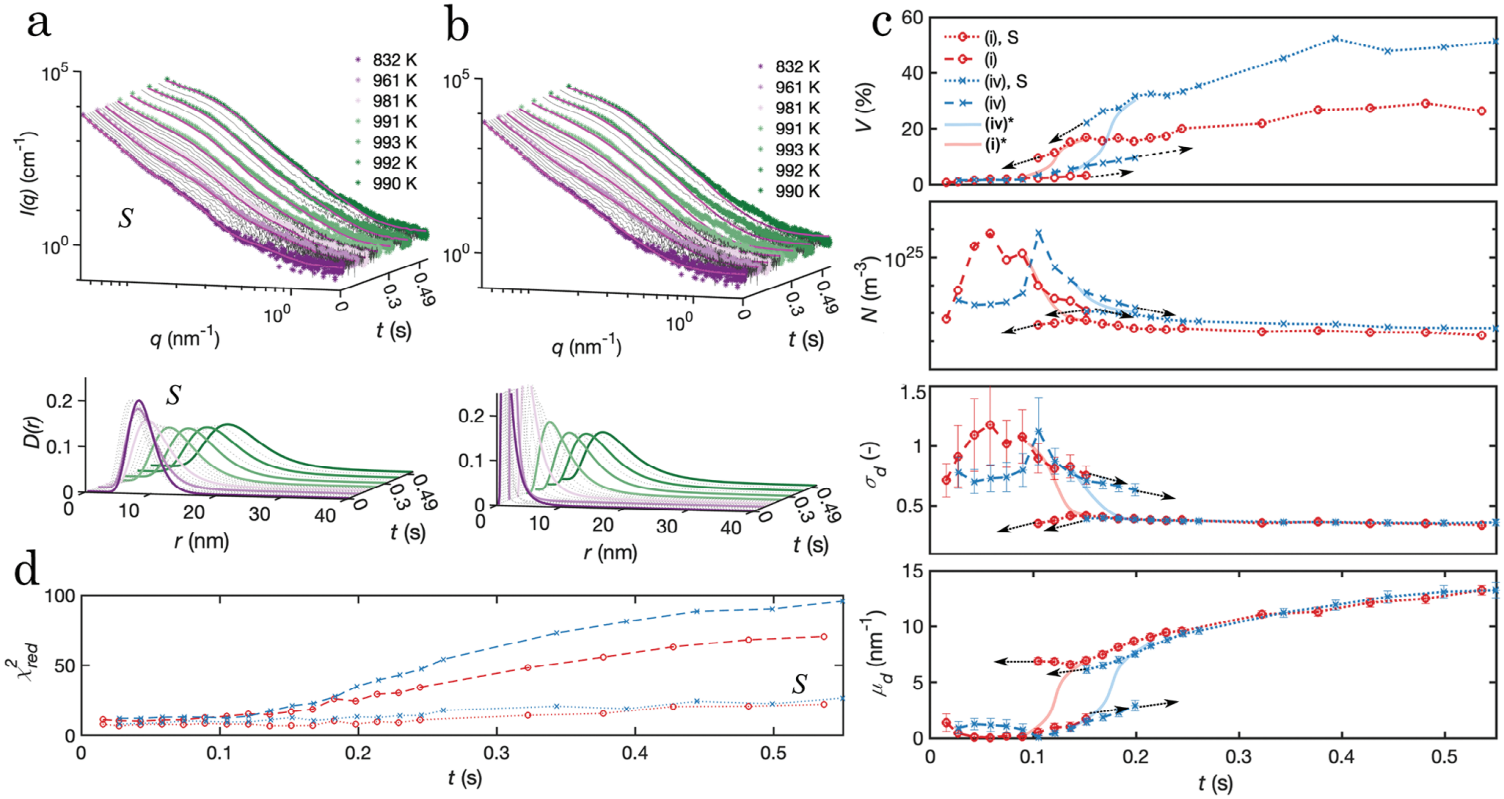
Crystalline phase evolution monitored by SAXS during flash‐annealing of sample (i) and (iv) with a current of 12A. a, b) from top to bottom: Selected absolute scaled SAXS patterns with the corresponding model fitting (red lines) and particle size distributions, including the use of a structure factor, *S*, or setting it to unity, extracted from sample (i), respectively. c) Selected ranges of the extracted parameters from the intensity model. From top: The total crystalline volume fraction, *V*, the number density of scattering particles, *N*, the width of the distributions, σ_*d*_, and the median particle size, µ_*d*_, from sample (i) and (iv) with the use of a structure factor and setting it to unity, respectively. Marked with red for sample (i) and blue for sample (iv) is a suggested transition ((i)* and (iv)*) between the two parameter sets, chosen in accordance with the main nucleation event and the reduced chi‐squared, $\chi_{red}^{2}$, in (d). The results presented in (a, b) for sample (iv), together with the full separated extracted parameter sets in (c), and the parameter data *S*, can be found in Experimental data analysis, Parameter extraction from SAXS pattern (Supporting Information).

In **Figure** **4**, results from the SAXS measurements that were recorded alongside with the WAXS measurements during devitrification of samples (i) and (iv) shown in Figures 1 and 2 are presented. As the SAXS technique depends on the scattering contrast, the analysis is limited to the vicinity of *t*_*x*1_ where the sample pieces have not devitrified to a point when the scattering contrast between matrix and precipitates becomes non‐representative. The SAXS signal *I*(*q*) exhibits a single peak in intensity, starting from a high scattering vector value at *q* = 0.9 nm^−1^ and decreases to *q* = 0.15nm^−1^ as the flash‐annealing process proceeds. Based on scanning electron microscope micrographs of partially crystallized AMLOY‐ZR01,^[^ ^8^ ^]^ a polydisperse log‐normal distribution, *D*(*r*, µ_*d*_, σ_*d*_), of spherical scattering particles is assumed.^[^ ^33^, ^34^ ^]^ Including background scattering, $c_{0}+c_{1}q^{-c_{2}}$, the scattered intensity can be modeled, by applying a local monodisperse approximation, with the function^[^ ^35^ ^]^

(1) $$\begin{array}{ccc} I(q) & = & N\Delta \rho^{2}\int_{0}^{\infty }S\left(q,r,\eta_{d},\alpha_{d}\right)D\left(r,\mu_{d},\sigma_{d}\right){\left\{V\left(r\right)F\left(q,r\right)\right\}}^{2}dr\\ & & +\;c_{0}+c_{1}q^{-c_{2}} \end{array}$$

The time dependent total scattering contrast, Δρ, is given by the sum of the contributing phases, β, and their corresponding crystalline phase weight fraction, *V*_β_, as Δρ = ∑_β_Δρ_β_ *V*_β_, where *V*_β_ is taken from the Rietveld refinement (as seen in Figures 1c and 2c). The scattering contrast is calculated using a stoichiometric approximation,^[^ ^36^ ^]^ yielding: 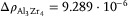 Å^−2^, 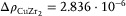 Å^−2^, and 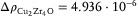 Å^−2^. From this we know that the extracted distribution will mostly be represented by the Al_3_Zr_4_ and Cu_2_Zr_4_O type structures but all contrasts are included in the fitting process. The structure factor, *S*(*q*, *r*, η_*d*_, α_*d*_), describes particle‐particle interactions that affect the scattered signal within the local volume η_*d*_, with particles of radius *r*α_*d*_ where α_*d*_ is a scaling factor.^[^ ^35^, ^37^ ^]^ For a dilute distribution of scattering particles, the structure factor should be considered as unity (*S* = 1), but as the devitrification proceeds the effect of the structure factor should be taken into account.^[^ ^37^ ^]^ The contribution to scattered intensity from a single homogeneous sphere with radius, *r*, and volume, *V*(*r*), is described by the form factor, *F*(*q*, *r*). The model that describes the intensity, Equation (1), was fitted to the experimental data to extract the parameters using a Matlab script, by separating the non‐linear and the linear terms as described in ref. [38].

The fitting of the model to selected SAXS patterns from sample (i) including the use of a structure factor (Figure 4a) and setting it to unity (Figure 4b) yields completely different PSD's. In Figure 4c, selected parts of the underlying parameters of these two sets of distributions extracted from samples from series (i) and (iv) together with the extracted number density, *N*, and the crystallized volume fraction, *V*, are presented. By evaluating the reduced chi‐squared, $\chi_{red}^{2}$, shown in Figure 4d, we see that after ≈ 150ms the parameters start to be better represented when the structure factor is included. This coincides with a decrease in the number density and distribution width, σ_*d*_, and an increase in median precipitate radius, μ_*d*_, (Figure 4c), which in turn correlates to a more growth dominated transformation mechanism occurring at higher temperatures (visualized by the reached temperature plateau in Figures 1a and 2a). A suggested transition between these two sets of results ((i)* and (iv)*) is indicated by a solid line for each sample. Data fitting with a model for small polydispersity was also attempted,^[^ ^37^ ^]^ but did not improve the goodness of the fit. Selected model fittings of sample (iv) together with the full sets of separated distribution parameters for the two samples are found, together with the parameter data of η_*d*_ and α_*d*_, in Experimental data analysis, Parameter extraction from SAXS pattern (Supporting Information).

Evaluating the suggested evolution of the parameters with the transition between the two sets, we see that both samples exhibit the same peak in number density, but shifted in time, which is reasonable considering how $t_{1}^{\Phi }$ is related to the nucleation event of the main phases. Sample (iv) exhibits a close to constant initial number density of ≈ 10^23^ m^−3^ during the first 80 ms. The likely origin of this is the formation of the Cu_2_Zr_4_O phase at $t_{1}^{\Phi }$, which occurs before the main nucleation event in sample (iv) (Figure 2b). These precipitates appear to trigger a more rapid transformation of the crystallized volume fraction compared to sample (i) and correlates with the relative weight phase fraction of the Cu_2_Zr_4_O phase (Figures 1c and 2c) in combination with the calculated scattering contrast of the different phases. Initially sample (iv) shows a slightly smaller width of the distribution and larger median radii of the clusters compared to sample (i), but after the main nucleation event (100–150ms) these parameters converge to the same magnitude. Similarly, after 200–300ms of structural clustering, the number densities also converge to the same magnitude of ≈ 10^22^m^−3^, which indicates that the growth mode starts to dominate the devitrification.

### 2.3 The Mode of Nucleation Affects Devitrification

The mechanisms governing the devitrification observed experimentally are numerically evaluated with homogeneous and heterogeneous devitrification simulations. For this, a model based on CNGT coupled to the calculation of phase diagrams (CALPHAD) methodology is used.^[^ ^39^ ^]^ The model originates from the work done by Ericsson and Fisk^[^ ^30^ ^]^ but has been further developed to better represent the interfacial energy between the amorphous matrix and the precipitating phases, incorporate heterogeneous and homogeneous nucleation, and account for different diffusion rates of the constituent elements in the matrix. The devitrification in the samples is simulated using the thermodynamic database for the ternary Al‐Cu‐Zr system developed by Zhou et al.^[^ ^32^ ^]^ Allowing the intermixing of Cu and Al with fixed composition of Zr, the Cu‐ and Al‐rich phases are modeled as line compounds; (Al,Cu)Zr_2_ and (Al,Cu)_3_Zr_4_. Excluding the contribution of Nb and O, this is consistent with the intermixing of Cu and Al observed from the EDS analysis in Figure 3 ($x_{{\text{CuZr}}_{2}}^{\beta }\approx {\text{Al}}_{0.05}{\text{Cu}}_{0.30}{\text{Zr}}_{0.55}$ and $x_{{\text{Al}}_{3}{\text{Zr}}_{4}}^{\beta }\approx {\text{Al}}_{0.20}{\text{Cu}}_{0.15}{\text{Zr}}_{0.50}$. Note that the sum is not unity, since the contributions from Nb and O are excluded). The Cu_2_Zr_4_O type structure is incorporated as heterogeneous cores because of the initial observed Bragg reflections attributed to this phase during the flash‐annealing of sample (iv) (Figure 2). By assuming a complete inclusion of a spherical particle with radius *r*_*core*_, the energy barrier for heterogeneous nucleation can be expressed as^[^ ^40^ ^]^

(2) $$\Delta G{\left(r\right)}_{het}=-\frac{4\pi (r^{3}-r_{core}^{3})}{3V_{m}^{\beta }}d_{c}+4\pi \left(r^{2}-r_{core}^{2}\right)\sigma \left(r\right)$$

where $V_{m}^{\beta }$ represents the molar volume of the precipitating particle, *d*_*c*_, the chemical driving force, and σ(*r*) is a radius dependent interfacial energy. With *r*_*core*_ = 0, we get the well‐known expression for a homogeneous energy barrier. Applying the energy barrier from Equation (2) at the critical radius for nucleation, r*, the steady‐state nucleation rate, *J*_ss_, is constructed as an Arrhenius type relation with k_B_ as the Boltzmann constant, expressed as^[^ ^40^ ^]^

(3) $$J_{\mathrm{ss}}=N_{0}Z_{f}k^{*}\text{exp}\left(-\frac{\Delta G(r^{*})}{k_{B}T}\right)$$

where *N*_0_ corresponds to the number of nucleation sites, *Z*_*f*_ is the Zeldovich factor, and k* is the condensation rate.

**Figure 5:**
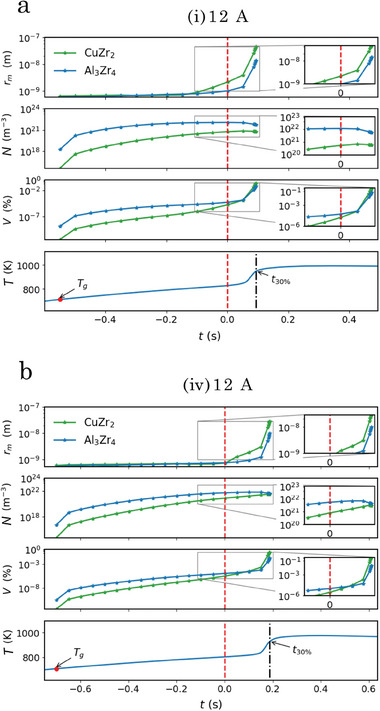
Evolution of distribution parameters during flash‐annealing simulations with temperature data from flash‐annealing experiments with a current of 12A. a,b) Crystalline volume fraction, *V*, the number density, *N*, the median radius, *r*_*m*_, and the temperature data that model is feed from *t*(*T*_*g*_) to $t_{30\%}$ for samples (i) and (iv) using homogeneous and heterogeneous nucleation modes, respectively. *t*_*x*1_ is marked with a dashed red line throughout the plots, and $t_{30\%}$ marks the time where 30% crystalline volume is reached and the simulations are terminated.

**Figure 6:**
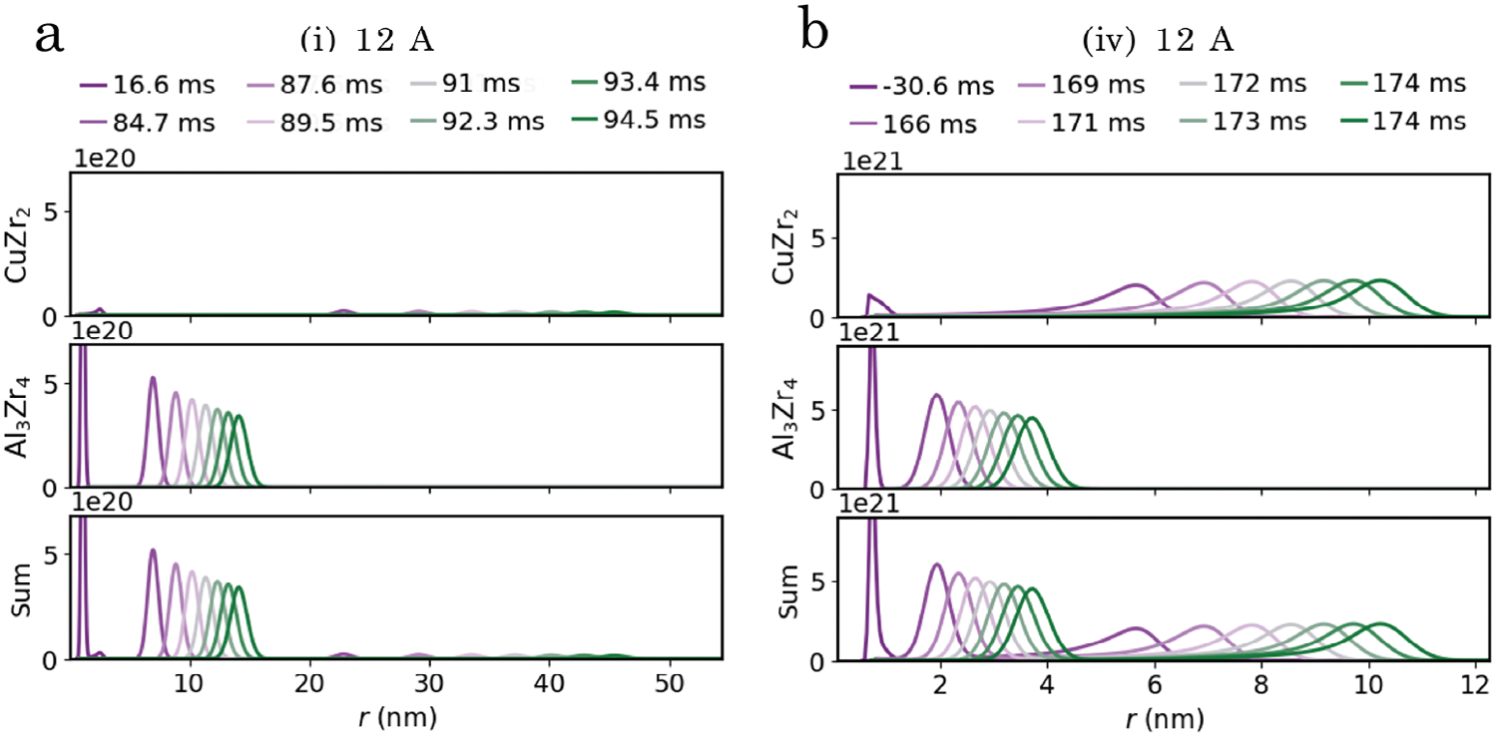
Number density distributions extracted from simulations with temperature data from flash‐annealing experiments with a current of 12A. a,b) The evolution of the number density distributions from homogeneous and heterogeneous simulations using the temperature feed from samples (i) and (iv), respectively. The first extraction is taken at 10^−4^% of total volumetric transformation and the last extraction is taken at $t_{30\%}$. The times are measured from the onset of devitrification.

The simulation results using the two nucleation modes coupled to the temperature data from samples (i) and (iv) seen in Figures 1a and 2a are presented in **Figures** **5** and **6**. The simulations were restricted to a total crystallized volume fraction of 30% since the formed particles grow without incorporating boundary interactions with other particles.^[^ ^41^ ^]^ The model captures an assumed homogeneous devitrification when all phases are formed concurrently, as seen in Figure 1c, and an assumed heterogeneous devitrification when one phase is formed before the other phases, as seen in Figure 2c. In the left part of Figure 5a,b the monitored parameters from the simulations are presented. The introduced cores, representing the Cu_2_Zr_4_O phase, increase the maximum of the parabolic nucleation rates of the CuZr_2_ and Al_3_Zr_4_ phases and shifts them to higher temperatures (Classical nucleation and growth theory, Modeling results, Supporting Information). This favors the nucleation of the CuZr_2_ phase more than the Al_3_Zr_4_ phase. As a result, this reduces the relative crystallized volume fraction of the Al_3_Zr_4_ phase in the heterogeneous case compared to the homogeneous (22% to 4%), which is consistent with the observed WAXS results, where we can observe a difference in the volume fraction of the Al_3_Zr_4_ phase in samples (i) and (iv). The suggested more rapid phase‐transformation observed in the SAXS analysis (Figure 4) is also reflected in the simulations where the simulation limit $t_{30\%}$ is reached at a slightly lower temperature with the heterogeneous implementation compared to the homogeneous nucleation mode. The observed increased nucleation rate confirms what Yang et al.^[^ ^25^ ^]^ reported, where the reduced thermal stability of AMLOY‐ZR01 was investigated using KJMA modeling. The temperature dependent steady‐state nucleation rates for the two different modes are given in Classical nucleation and growth theory, Modeling results (Supporting Information).

The solubility in the simulated phases was evaluated using the particle compositions as a function of the cluster radii at the end of the simulations, extracted from the growth function under the local equilibrium condition. A slight composition change is observed at small radii while the composition is close to constant for cluster radii larger than 10nm. Samples (i) and (iv) exhibit minor differences in particle composition, but both give an intermixing of Al and Cu; $x_{{\text{CuZr}}_{2}}^{\beta }={\text{Al}}_{0.06}{\text{Cu}}_{0.27}{\text{Zr}}_{0.67}$ and $x_{{\text{Al}}_{3}{\text{Zr}}_{4}}^{\beta }={\text{Al}}_{0.32}{\text{Cu}}_{0.11}{\text{Zr}}_{0.57}$. These results are comparable to what is observed in the EDS micrographs in Figure 3. More information is given in Classical nucleation and growth theory, Modeling results (Supporting Information).

In Figure 6, the simulated number density distributions from a homogeneous nucleation mode in sample (i) and a heterogeneous nucleation mode in sample (iv) are presented. The major transformation occurs during the last 5–10ms of the simulations when the temperature starts to favor the growth of the particles. The introduced cores result in distributions with lower median radii but higher number densities compared to the homogeneous case. The lower median radii is something that we do not observe from the SAXS analysis, but we can see that the median radii of the Al_3_Zr_4_ distribution is between the two sets of results obtained in Figure 4c. We stress however that what is seen in the SAXS analysis is mostly representative of Al_3_Zr_4_ and Cu_2_Zr_4_O whereas the simulation results represent the total transformed fraction. The HAADF STEM micrographs of the devitrified end product of samples (i) and (iv) (Experimental data analysis, Microstructural analysis with TEM, Supporting Information) does suggest this trend where the dark regions rich in Al are smaller in sample (iv) compared to sample (i). Furthermore, our modeling results give information about the distribution of the CuZr_2_ phase, which is mostly obscured in the SAXS patterns. We see that for both nucleation modes, this distribution consists of fewer but larger particles compared to the Al_3_Zr_4_ phase.

### 2.4 Structural Changes During Melting and Solidification

**Figure 7:**
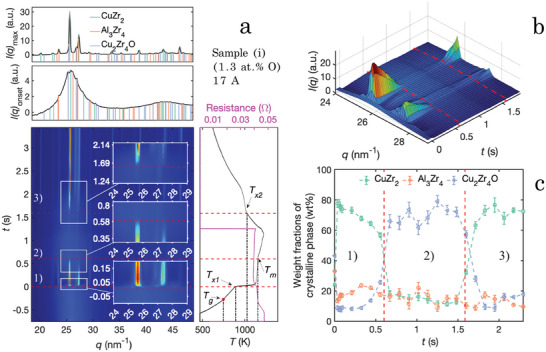
Structural phase ordering and disordering in sample (i) containing 1.3at.% O during flash‐annealing with a current of 17A resulting in an approximated linear heating rate Φ_*lin*_ = 800Ks^−1^. Top (a): Diffraction pattern at the maximum intensity during devitrification and at the onset of devitrification, *t*_*x*1_. Bottom (a): Integrated WAXS patterns from the flash‐annealing measurements. It consists of three events: 1) devitrification when 0 < *t* < *t*_*m*_, 2) dissolution when *t*_*m*_ < *t* < *t*_*x*2_ and, 3) crystallization when *t*_*x*2_ < *t*. The insets show close ups of the events. Right bottom part of (a): Time dependent temperature curves (black) and resistivity curves (pink). A sharp increase in temperature is observed at the exothermic nucleation event. The melting temperature *T*_*m*_, the onset of crystallization *T*_*x*2_, the onset of devitrification *T*_*x*1_, and the glass transition temperature *T*_*g*_, are marked with dashed black lines. b) A 3D representation of the dissolution and crystallization events where the red dashed lines represent the onset of the events. c) Weight phase fraction of the crystalline volume obtained by Rietveld refinements of the WAXS patterns, starting from devitrification with *t*_*m*_ and *t*_*x*2_ are marked with red dashed lines.

In **Figure** **7**, the diffraction results from in situ flash‐annealing of sample (i), heated with a constant current of 17A for 2s (resulting in an approximated linear heating rate Φ_*lin*_ = 800Ks^−1^) are presented. For this measurement sequence, the relatively high current was enough to melt the longitudinal center of the sample within the short time it was supplied. The heat generated in the sample first triggered a rapid devitrification of the glass phase, which was then followed by partial dissolution of the Al_3_Zr_4_ and CuZr_2_ phases when the temperature in the sample reached the onset melting temperature, *T*_*m*_, at time *t*_*m*_. All phases remain during melting, but the Cu_2_Zr_4_O phase is dominating the crystalline weight fraction. When the current is turned off, a long‐range ordering with an onset time *t*_*x*2_, and a temperature *T*_*x*2_, is observed during the quenching process (≈ 430Ks^−1^). The onset of melting is extracted when an apparent loss of signal from the main phases is observed and, subsequently, the onset of crystallization is extracted when the signal from these phases emerges once again. The same trends are seen in all samples subjected to an annealing current of 17A regardless of oxygen content, however, the intensity of the reflections attributed to the Cu_2_Zr_4_O phase varies with oxygen content. The results of sample (iv), of the matching process shown for sample (i) in Figure 7, are shown in Experimental data analysis, Structural changes during melting and solidification in sample (iv) (Supporting Information).

During the crystallization event (*t* ⩾1500ms), the CuZr_2_ phase rapidly grows and becomes the largest contribution of the crystalline phase after 200ms and begins to stabilize after 300ms. When comparing the crystallization process with the devitrification in sample (i), subjected to a flash‐annealing with a current of 12A (Figure 1), the formation of the phases does not occur simultaneously, instead the Al_3_Zr_4_ phase is formed after the CuZr_2_ phase. The reason for this is believed to correlate with heterogeneous nucleation induced by the Cu_2_Zr_4_O phase, since that route favors the CuZr_2_ phase over the Al_3_Zr_4_ phase (Figures 1 and 2), and/or changes in the temperature‐dependent chemical driving forces. These results are in line with what Yang et al.^[^ ^5^ ^]^ reported to be the main mechanism behind crystallization from the cyclic reheating during AM processing of AMLOY‐ZR01. The growth of the crystalline phases strongly correlates with the amount of quenched‐in nuclei, which in the reported case was related to the oxygen concentration in the sample during casting.

**Figure 8:**
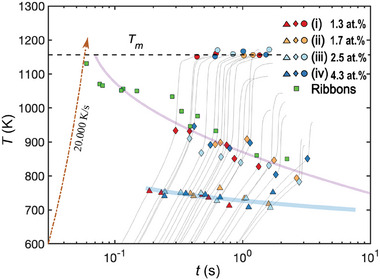
The CHT diagram produced from the scattering measurements. Extracted devitrification onset time and temperatures *t*_*x*1_ and *T*_*x*1_ (marked as rhombus) together with the glass transition temperatures *T*_*g*_ (marked as triangles), from the samples (i)‐(iv). The melting temperature *T*_*m*_ is extracted from the DSC‐measurements (mean temperature of samples (i)‐(iv)). The melting temperature *T*_*m*_ from the X‐ray experiments are shown as circles. The temperature curves from the flash‐annealing experiments are included for visualization. The data from the ribbons show the onset of devitrification at higher heating rates (marked as green squares), but the temperature curves and glass transition temperatures are excluded because of the limiting resolution in this data. The light purple and blue lines are guides for the eye.

In **Figure** **8**, a CHT diagram is presented. Data points corresponding to (*T*_*g*_, *t*_*g*_), (*T*_*x*1_, *t*_*x*1_), and (*T*_*m*_, *t*_*m*_) from 22 flash‐annealing experiments on sample pieces from the four series with constant currents of 10,12,15,17,20,25A are shown, including the experimental results of samples (ii) (1.7at.% oxygen) and (iii) (2.5at.% oxygen), as well as previously mentioned samples (i) and (iv). In addition to the suction cast samples, the CHT also includes data from melt‐spun ribbons which was collected using a similar setup.^[^ ^31^ ^]^ The ribbons were manufactured from an alloy with the same composition as sample (i), but had a smaller cross‐section and could therefore reach a higher heating rate compared to samples (i)‐(iv). This makes it possible to access the lower part of the nose in the CHT diagram, where the critical approximated linear heating rate is estimated to be 2.0· 10^4^Ks^−1^. The heating rates in the ribbon‐annealing experiment were so high that the resolution of the temperature data was insufficient to extract *T*_*g*_ and *t*_*g*_. For this reason, only onset of devitrification temperature and time (*T*_*x*1_, *t*_*x*1_) were extracted for the ribbons.

The estimated critical heating rate is of the same magnitude as what has been observed in previous studies of AMLOY‐ZR01.^[^ ^25^, ^26^ ^]^ From CHT and time temperature transformation (TTT) diagrams produced with FDSC, a critical heating rate was reported to be 4.5· 10^4^Ks^−1^.^[^ ^26^ ^]^ From another study where the same measuring technique was used on powder containing oxygen concentrations comparable to sample (i),^[^ ^25^ ^]^ the critical heating rate was estimated to 2.5· 10^4^Ks^−1^ from the produced TTT diagram. The extracted data from samples (i)‐(iv) shows a spread, but no trend relating to the different oxygen levels can be observed. Hence, in relation to previous studies showing how the thermal stability of the alloy is reduced with increasing oxygen content,^[^ ^5^, ^7^, ^25^ ^]^ no further reduction is observed in the concentration range 1.3–4.3at.%. However, from our measurements we see that the devitrified end product is altered.

## 3 Discussion

In this work, we present uniquely detailed descriptions of the phase evolution in Zr‐based bulk metallic glass samples subjected to flash‐annealing by Joule heating with constant currents in the range of 10–25A monitored in situ with WAXS and SAXS measurements carried out with a time resolution of 4ms. With additional ribbons, approximated linear heating rates in the order of 10^2^–10^4^Ks^−1^ are obtained. The composition of the samples is based on the commercially available alloy AMLOY‐ZR01^[^ ^21^ ^]^ (Zr_59.3_Cu_28.8_Al_10.4_Nb_1.5_) with varied amount of added oxygen (1.3 – 4.3at.%). By using CNGT, the observed devitrification was successfully modeled and phase specific information were extracted. The microstructure of the devitrified end product was analyzed with TEM and the composition of the samples was obtained using ToF‐ERDA.

Regardless of the heating rate and oxygen concentration in the sample, all three identified phases (CuZr_2_, Al_3_Zr_4_, Cu_2_Zr_4_O) are present in the devitrified and/or crystallized end product. The CuZr_2_ phase is the dominating phase in the transformed end product. Our results show that the oxygen‐induced Cu_2_Zr_4_O phase remains at temperatures above the melting temperature, *T*_*m*_, whereas the other observed phases almost completely dissolve (Figure 7). This is consistent throughout our samples regardless of the oxygen concentration. The intensity of the diffraction peaks corresponding to this phase is, however, dependent on the oxygen concentration, implying that the amount of Cu_2_Zr_4_O is varying with the concentration. The observed higher thermal stability of the Cu_2_Zr_4_O phase (Figure 7) implies that, if oxygen contamination is present, the temperature gradient in the close vicinity of the melt pool during AM processing of material would be susceptible to quenched‐in nuclei, which would further grow in the HAZ upon continuous processing. This is of great interest to the AM community because it explains in detail the underlying problems that are related to the cyclic remelting during processing. For example, the mechanism behind the observed quenched‐in nuclei within the HAZ^[^ ^5^, ^8^ ^]^ after AM processing of Zr‐based material, correlated to the oxygen concentration, is by this clearly explained.

During AM the atmosphere in the chamber is not the same as in this study, but in principle this should not impact the parallels drawn to this processing method. It is hard to separate oxygen contamination in the powder feedstock material from additional contamination from the atmosphere in the building chamber. Therefore, our focus has been on the observed concentration in the end product rather than on the source of the contamination. In our samples, no trends are observed between the onset of devitrification and oxygen concentration (Figure 8), suggesting that the thermal stability of the alloy is not further reduced by increasing the oxygen concentration from 1.3 to 4.3at.%. This should in principle be of significant importance, indicating that powder handling costs and protective gas requirements could be reduced if oxygen contamination is apparent during manufacturing. However, our results reveal that elevated oxygen levels >2at.% alter the phase fraction hierarchy during devitrification, and the volume of the hard Cu_2_Zr_4_O ($Fd\bar{3}m$) phase seems to correlate with the concentration. As this phase has been shown to induce the opposite effect^[^ ^11^, ^19^ ^]^ of reinforcement by ductile nano‐particles in MGCs,^[^ ^15^, ^16^, ^17^, ^18^ ^]^ the reduction of oxygen impurities is alas critical for the Zr‐based glass forming systems.

From the analysis of our data, it is for the first time shown that heterogeneous nucleation becomes the dominant nucleation mechanism during devitrification at oxygen levels >2at.%. The implemented heterogeneous CNGT model impact the nucleation rate of the CuZr_2_ phase the most, and the obtained simulation results are in line with the weight phase fractions presented in Figures 1c and 2c, where the relative volume fraction of CuZr_2_ to Al_3_Zr_4_ changes from a factor of four to six. This shows that the implemented heterogeneous nucleation mode is applicable when modeling the observed events. We have here presented not only strong proof for the previously suggested oxygen‐induced heterogeneous nucleation mechanism in Zr‐based BMG,^[^ ^14^, ^20^, ^23^, ^25^, ^42^ ^]^ but also shown how, and in what way, this impacts the specific phases during the rapid transformations. In relation to the development of MGC this is of significant importance as the introduction of nano‐particles in a BMG alters the conditions for phase formation. This could be utilized to broaden the composition ranges of interest, but it could also reduce this range, depending on how the mechanism impacts the phase formation. At oxygen concentrations <2at.% our results show that all phases are formed simultaneously, however, similar dendritic growth as observed in Figure 3a has also been observed in Zr_65_Al_7.5_Cu_17.5_Ni_10_ metallic glass^[^ ^23^ ^]^ where it originated from heterogeneous growth of CuZr_2_ on a core of NiZr_2_ ($Fd\bar{3}m$) phase. Nevertheless, even if traces of heterogeneous nucleation might, and is likely still present at these concentrations, the major mechanism for precipitation is here shown to be of homogeneous nature.

By performing SAXS measurements alongside WAXS measurements, statistical data of the particle size distribution in the bulk material could be extracted. Furthermore, the synergy that this technique has with CNGT makes this a preferable approach for these types of studies. By extending the scattering analysis with numerical simulations, phase specific distributions were acquired that would otherwise be extremely hard to capture, if not impossible. From the high quality scattering data we have observed how the rapid event of devitrification unfolds, as well as how the kinetics of phase formation is changed upon quenching. We have captured the rapid nucleation event with both SAXS and WAXS where the time resolution was critical for proper evaluation of the event. The high signal‐to‐noise ratio made it possible to distinguish minor diffraction peaks and the effect of small composition variation, resulting in unprecedented description of the phase evolution extracted through Rietveld refinements. In the SAXS data, this was also the key to distinguish scattering intensity from small volumes of precipitates of small radii. Thus, the utilization of cutting edge experimental equipment provides important tools necessary for the understanding the characteristic features of glass forming alloys during rapid phase transformations.

## 4 Conclusion

In summary, we have used a combination of in situ scattering measurements together with numerical simulations to investigate phase evolution during flash‐annealing of samples of AMLOY‐ZR01 containing different amounts of oxygen. Additionally, to characterize the composition and nano‐structure, TEM measurements were performed on selected samples. This multi‐technique approach provides in‐depth analysis and thorough mapping of phase evolution in glassy alloys. During devitrification, the phase fraction hierarchy strongly correlates with oxygen concentration. Simulations with a homogeneous and heterogeneous nucleation mode capture the experimental observations and provide phase specific particle size distributions. During dissolution, the oxygen rich phase becomes the dominant crystalline phase. From the extracted critical temperatures and times, a continuous heating transformation diagram was constructed where no further reduction of the glass forming ability was observed at elevated oxygen concentrations. The results presented should be of broad relevance as the crystalline phases that form upon devitrification are often similar among the Zr‐based glass‐forming alloys. Furthermore, the methodology shows great promise for evaluating other glass forming systems where a controlled amount of precipitates is of interest. Material properties such as ductility, corrosion resilience, and magnetic properties are features that can be tuned with a dispersion of nano‐particles in BMG, and the tools that we have provided in this study give the means to measure and identify these distributions.

## 5 Experimental Section

### Sample Preparation

Two batches of samples were prepared in the present study: one set of suction cast bulk metallic glass samples with varying oxygen content (samples (i)–(iv)) and one set of ribbons. The aim of producing two sets of samples with different thickness was to be able to vary the heating rates during the flash‐annealing studies.

The cast samples were produced from four alloys with controlled oxygen content and nominal compositions of Zr_59.3 − *x*_Cu_28.8_Al_10.4_Nb_1.5_O_*x*_ with x = 0, 0.2, 0.4, 0.6 that were weighed in from high purity raw elements (Alfa Aesar): Zr crystal bar (Oxygen < 50 ppm, 99.5% (metals basis)), Cu slugs (Oxygen free, 99.997%), Al (99.99% (metals basis)), Nb slugs (61 ppm oxygen, 99.992%) and subsequently arc‐melted. Oxygen was introduced to the composition by adding CuO in powder form (Cerac, 99.9%). To ensure the incorporation of the powder into the melt during arc‐melting, the CuO was wrapped in Cu foil (Alfa Aesar, 99.9999% (metals basis)). The arc‐melting of the alloys was performed in Ar‐atmosphere after melting a Ti‐getter multiple times to purify the atmosphere. The samples (rods of 3mm diameter and about 50mm long) were obtained by suction casting using an arc‐melting furnace (Edmund Bühler GmbH) with an attached Cu mold. The furnace was evacuated to 3· 10^−5^mbar and back‐filled with Ar to a pressure of 600mbar. In addition, a Ti‐getter was used to absorb residual oxygen in the furnace. The melt was deposited into the Cu mold by a pressure difference of at most 600mbar upon triggering the suction casting. The final sample pieces (approximately ten for each oxygen concentration) were cut from the rods as rectangular sheets with dimensions 10 × 1 × 0.5mm by electrical discharge using an Agie Charmilles FI 440 ccS instrument from GF Machining Solutions. After grinding, the thickness of the samples was ≈0.46 mm.

Ribbons with the nominal composition of Zr_59.3_Cu_28.8_Al_10.4_Nb_1.5_ were produced from a master ingot composed of high‐purity elements (> 99.98%) by arc melting under Ti‐gettered Ar (99.999%) atmosphere. Glassy ribbons (≈ 40µ m thick, ≈ 1.4mm wide) were prepared by using the melt‐spinning technique at ≈ 1300K ejection temperature and 29ms^‐1^ Cu wheel rotating speed. The final samples were cut into ≈ 5.4cm long pieces.

### Sample Characterization

In order to determine the composition of an as‐synthesized sample from each of the cast samples, ToF‐ERDA was performed at the beamline of the Tandem Laboratory at Uppsala University.^[^ ^43^ ^]^ The system used a combination of a gas ionization chamber (GIC), as well as time of flight detectors giving information about both the elemental species and their energy level, which provided a fine depth resolution. The measurements were done with an incident beam of ^127^ *I* ^10+^ and an energy of 44 MeV. The setup had a standard geometry with the detector at 45° and the sample at 67.5° and the data analysis was performed using Potku^[^ ^44^ ^]^ with the efficiency files supplied by Tandem. Together with the four samples, a reference sample for energy calibration and a sapphire screen used for positioning of the beam spot were mounted on the sample holder. See **Table** **1** for the measured composition. The composition and statistical error were calculated from the integrated profile corresponding to the bulk of the sample, below the surface oxide (<100nm). The statistical error was based on the number of events corresponding to each concentration in the chosen depth interval.^[^ ^44^ ^]^ The acquired data used for the composition analysis can be found in Experimental data analysis, Composition analysis with ToF‐ERDA (Supporting Information).

**Table 1:** Sample composition derived from ToF‐ERDA experiments, and melting temperatures extracted with DSC. It was not possible to separate the concentration of Nb and Zr because of their small difference in atomic mass.

Sample	Zr+Nb [at.%]	Cu [at.%]	Al [at.%]	O [at.%]	*T*_*m*_ [K]
(i)	56.6 ± 0.6	29.0 ± 0.5	13.2 ± 0.4	1.3 ± 0.1	1157 ± 3
(ii)	56.3 ± 0.5	29.2 ± 0.4	12.7 ± 0.3	1.7 ± 0.1	1155 ± 3
(iii)	55.4 ± 0.5	29.1 ± 0.4	13.0 ± 0.3	2.5 ± 0.1	1158 ± 3
(iv)	54.8 ± 0.5	29.0 ± 0.4	11.9 ± 0.3	4.3 ± 0.2	1154 ± 3

The melting temperatures of the cast samples were extracted from differential scanning calorimetry (DSC) measurements on one piece from each sample series. The scans were performed in an Ar‐flow with a heating rate of 20K min^−1^ up to 1000 °C, using NIETZSCHs simultaneous TG‐DTA/DSC apparatus, STA 449 F1 Jupiter. See Table 1 for the melting temperatures. The resulting profiles are found in Experimental analysis (Supporting Information).

TEM at 200kV accelerating voltage with energy dispersive X‐ray spectroscopy (FEI Titan Themis with SuperX EDS) was used to investigate a lamella prepared by focused ion beam milling (ZEISS CrossBeam550) from the center of sample (i) flash‐annealed with the current of 12A. SAED and nanobeam diffraction were employed to gather structural information from selected areas. EDS data processing was conducted with Hyperspy.^[^ ^45^ ^]^ Diffraction data was analyzed with the support of CrysTBox.^[^ ^46^ ^]^

### In Situ Scattering Measurements

Combined SAXS and WAXS measurements during flash‐annealing of samples (i)‐(iv) were carried out at beamline P21.2, Petra III, Desy, Hamburg^[^ ^47^ ^]^ with a photon energy of 52.7keV. The SAXS pattern was recorded using a PILATUS3 X CdTe 2M detector positioned at the distance of 14.6m from the sample. A vacuum tube was mounted between the detector and the sample to minimize noise. An EIGER X 4M detector positioned at the distance of 0.876m from the sample was used to record the WAXS pattern. The WAXS and SAXS detectors were calibrated using a CeO_2_ and a AgBh sample, respectively. The sampling frequency used for both detectors was 250Hz.

Additional WAXS measurements during flash‐annealing of glassy ribbons were performed at beamline P21.1, Petra III, Desy, Hamburg^[^ ^47^ ^]^ with a photon energy of 101.4keV. The WAXS patterns were recorded by using a PILATUS3 X CdTe 2M detector positioned at a distance of 0.993 m from the sample. The WAXS detector was calibrated using a LaB6 sample. The sampling frequency was 250Hz.

Flash‐annealing resistive Joule heating setups^[^ ^31^ ^]^ were used for both batches, which allowed for in situ scattering measurements during the devitrification, melting and crystallization events. During the measurements, the vacuum in the flash‐annealing setups was kept at ≈10^−5^mbar. With a Delta Elektronika SM 52–30 power supply, the samples were heated with a constant current mode by passing the current through the samples, resulting in a temperature maximum in the longitudinal center of each sample. Through the sample thickness, the temperature was assumed to be homogeneous. A LumaSense IGA 320/23‐LO pyrometer was used to record the surface temperature of the samples with a resolution of 4Ks^−1^. The error of the pyrometer is 0.3% of the measured value. The temperature and time were coupled to the diffraction pattern using a trigger signal that relates the measured resistivity to the known beam exposure time. The temperature data was calibrated using the melting temperature extracted from DSC measurement of the samples and the lower detection limit of the pyrometer (423K).

### Rietveld Refinement and SAXS Data Extraction

Selected diffraction patterns were evaluated using the Rietveld method, in order to determine which phases were present and how they evolve with time under temperature. The program used for this evaluation was Fullprof.^[^ ^48^ ^]^ In order to establish the ratios between the crystalline phases, the amorphous part of the diffraction pattern was treated as a part of the background, using linear interpolation. The peak shape of the crystalline part of the patterns was then described using a Thompson‐Cox‐Hastings pseudo‐Voigt function with axial divergence asymmetry. The refined parameters for each pattern were the zero point, scale factors, unit cell parameters (a, b, c), half‐width parameters (U, V, W), peak shape variable (X), and the overall isotropic temperature parameters (B). The phases that had been reported in literature were evaluated and the three main phases Cu_2_Zr_4_O ($Fd\bar{3}m$), CuZr_2_ (*I*4/*mmm*), and Al_3_Zr_4_ (*P*6/*mmm*) in combination fitted well with the measured patterns in terms of representing the vast majority of the diffraction peaks in all patterns. Two minor peaks that in addition appear only in some of the patterns were not described with these phases. One of these residual peaks (*q* ≈ 28nm^−1^) could possibly belong to the Heusler phase AlCu_2_Zr ($Fm\bar{3}m$), which had the same space group as the reported Al_7_Cu_16_Zr_6_ phase.^[^ ^5^ ^]^ This phase was also mentioned to be one of the phases with the highest chemical driving force for the ternary Al_10_Cu_30_Zr_60_ system.^[^ ^30^ ^]^ However, because this was the only peak possibly correlating to that phase, it was deemed to be a to weak basis to confirm this phase and it was excluded from the refinement. The other reported phases (Al_2_Zr_3_^[^ ^6^ ^]^ and α‐Zr^[^ ^7^ ^]^) were also investigated as possible candidates, but did not overlap with the diffraction peaks of the pattern in such a way that it would improve the fit, and were thus excluded from further analysis.

To extract physically coherent parameter data from the SAXS measurements, the integrated intensity was first scaled to absolute values using a NIST SRM 3600^[^ ^49^ ^]^ sample following the procedure outlined by Allen et al.^[^ ^50^ ^]^ At sufficiently high X‐ray energies, the absorption edges were irrelevant, and the atomic scattering factors converge to the atomic number *Z*.^[^ ^36^ ^]^ To account for the relativistic behavior of the scattering length density (SLD), a correction factor was suggested such that Z* = *Z* − (*Z*/82, 5)^2.37^. This correction to *Z* did only contribute moderately for heavier elements. With the molecular volume, *V*_*mo*_, the SLD was given by $\text{SLD}=\frac{\sum_{i=1}^{n}Z_{i}^{*}}{V_{mo}}$ and the scattering contrast was given by the difference of scattering length density between the precipitate β and the matrix α (Δρ = SLD_β_ − SLD_α_).

### Precipitation Model

The molar enthalpy of fusion for the constituent elements were taken from Ref. [40] as $\Delta H_{m}^{f}$ (Al) = 10.5kJmol^−1^, $\Delta H_{m}^{f}\left(\text{Cu}\right)=13.0$ kJmol^−1^ and $\Delta H_{m}^{f}\left(\text{Zr}\right)=20.1$ kJmol^−1^. The distance to the nearest neighbor was taken as r_1_ = 3.05Å.^[^ ^51^ ^]^ The scaling factor to the number of nucleation sites relating to the heterogeneous nucleation was set to Γ = 10^−3^ and the diffusion rates of the constituent elements were scaled with the effective diffusion coefficient according to their atomic weight, setting Al as one. To overcome the non‐representative nucleation rate in the Al_10_Cu_30_Zr_60_ observed from the Al_3_Zr_4_ phase in the work by Ericsson and Fisk,^[^ ^30^ ^]^ the factor Π = 0.83 was used to scale the interfacial energy for this phase. *r*_*core*_ was taken as half of the smallest computed critical radius for the simulated phases, thus allowing the cores to grow during the simulations. To account for the implemented Einstein‐Stokes diffusion, as well as to better reflect the actual devitrification process, the starting temperature was set to *T*_*g*_. For a full description of the nucleation model and complementing phase specific simulation parameters, see Classical nucleation and growth theory (Supporting Information).

## Conflict of Interest

The authors declare no conflict of interest.

## Supporting information

Supporting Information

Supporting Information

## Data Availability

The data that support the findings of this study are available from the corresponding author upon reasonable request.
