# Supplementary material for: In Situ Mapping of Phase Evolutions in Rapidly Heated Zr‐Based Bulk Metallic Glass with Oxygen Impurities
Source: Adv Sci (Weinh). 2024 Feb 28;11(16):2307856. doi: 10.1002/advs.202307856 (PMC11040349; doi:10.1002/advs.202307856)
Supplement: Supplementary file 1 — Supporting Information [file ADVS-11-2307856-s001.pdf]

ADVANCED SCIENCE
Open Access

Supporting Information

for *Adv. Sci.*, DOI 10.1002/advs.202307856

In Situ Mapping of Phase Evolutions in Rapidly Heated Zr-Based Bulk Metallic Glass with Oxygen Impurities

Mattias Tidefelt, Julia Löfstrand, Inga K. Goetz, Olivier Donzel-Gargand, Anders Ericsson, Xiaoliang Han, Petra E. Jönsson, Martin Sahlberg, Ivan Kaban and Martin Fisk*

In situ Mapping of Phase Evolutions in Rapidly Heated Zr-Based Bulk Metallic Glass with Oxygen Impurities

Mattias Tidefelt, Julia Löfstrand, Inga K. Goetz, Olivier Donzel-Gargand, Anders Ericsson, Xiaoliang Han, Petra E. Jönsson, Martin Sahlberg, Ivan Kaban, Martin Fisk*

Supporting Information: Experimental data analysis

Complementing results not presented in the main article from the composition analysis, DSC measurements, TEM analysis, and SAXS/WAXS data analysis are presented here.

Composition analysis with ToF-ERDA. The results of the ToF-ERDA measurements are shown in their entirety below in Figures S1-S4. The right graph shows the data and cut out profiles and the left graph shows the resulting depth profiles and the region that make out the basis from where the average is taken as the bulk composition.

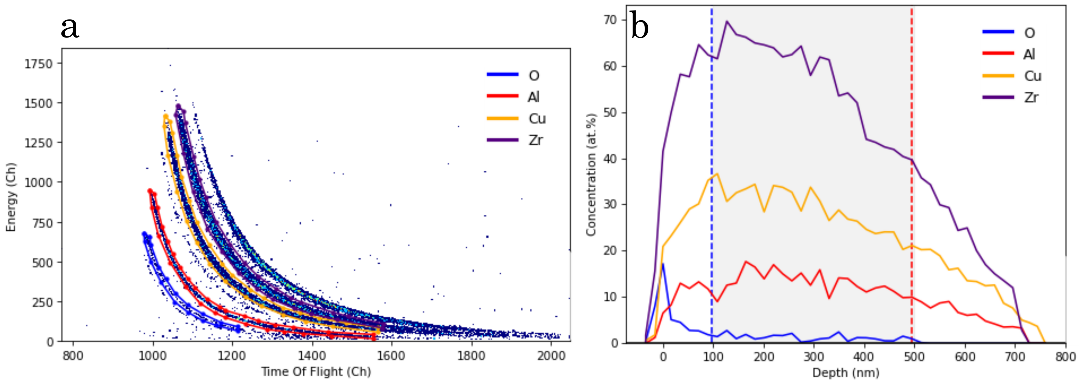

Figure S1: ToF-ERDA results for series (i), showing the (a) incidence maps and (b) corresponding depth profiles of the elements Zr, Cu, Al, and O. The composition of each element is based on the selected region shown in the depth profile chosen well beneath the surface oxide so it represents the bulk composition of the sample.

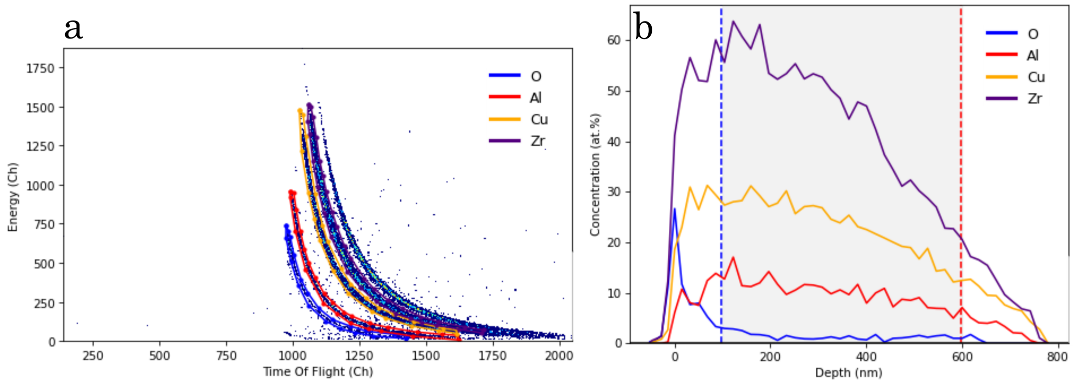

Figure S2: ToF-ERDA results for series (ii), showing the (a) incidence maps and (b) corresponding depth profiles of the elements Zr, Cu, Al, and O. The composition of each element is based on the selected region shown in the depth profile chosen well beneath the surface oxide so it represents the bulk composition of the sample.

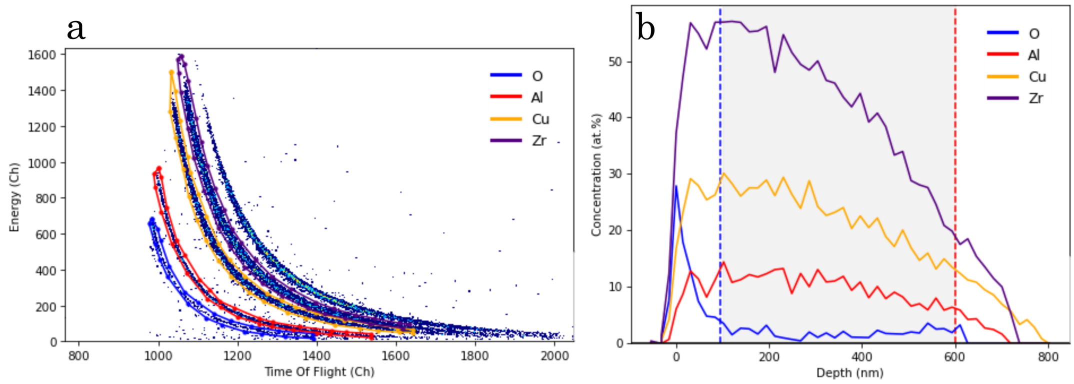

Figure S3: ToF-ERDA results for series (iii), showing the (a) incidence maps and (b) corresponding depth profiles of the elements Zr, Cu, Al, and O. The composition of each element is based on the selected region shown in the depth profile chosen well beneath the surface oxide so it represents the bulk composition of the sample.

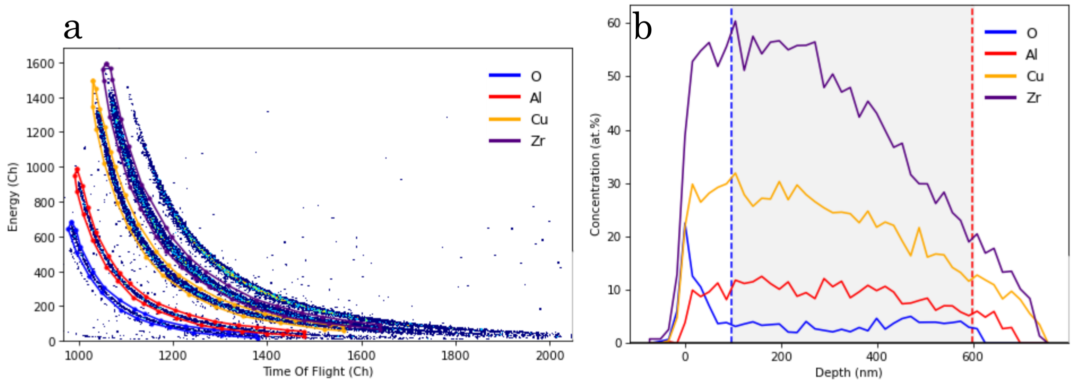

Figure S4: ToF-ERDA results for series (iv), showing the (a) incidence maps and (b) corresponding depth profiles of the elements Zr, Cu, Al, and O. The composition of each element is based on the selected region shown in the depth profile chosen well beneath the surface oxide so it represents the bulk composition of the sample.

Differential scanning calorimetry The resulting profiles from the DSC measurements are shown in Figure S5.

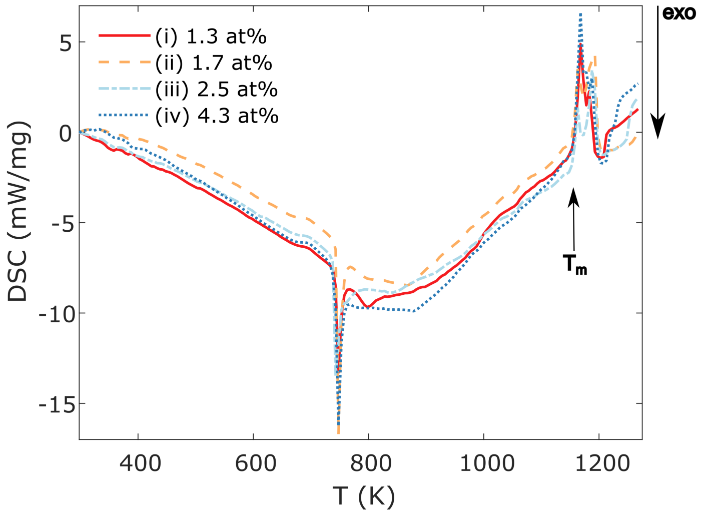

Figure S5: DSC scans from Room Temperature up to 1274 K for suction cast samples from each series (i)-(iv). With the heating rate 20 K/min the samples have a similar thermal response. The onsets of melting (T_m) are indicated with an arrow.

Approximated linear heating rates The approximated linear heating rates, Φ_{lin}, (room temperature to crystallization onset extracted from the temperature data) for all measurements on bulk samples are presented in Table S1.

Table S1: Approximated linear heating rates, Φ_{lin}, for samples (i)-(iv) obtained from the flash-annealing experiments with target currents: $10, 12, 15, 17, 20, 25\,\mathrm{A}$.

Sample	Current [A]	Φ_{lin} [Ks^{-1}]	Mean Φ_{lin} [Ks^{-1}]
(ii)	10	190	180
(iii)		180	
(iv)		170	
(i)	12	400	330
(ii)		310	
(iii)		340	
(iv)		280	
(i)	15	650	580
(ii)		570	
(iii)		570	
(iv)		530	
(i)	17	800	820
(ii)		900	
(iii)		830	
(iv)		740	
(i)	20	1660	1230
(ii)		980	
(iii)		1210	
(iv)		1070	
(i)	25	2150	1780
(iii)		1610	
(iv)		1570	

Phase ordering sequence at the onset of devitrification. In Figures S6 and S7, 3D visualization in the vicinity of the onset time t_{x1} of the WAXS measurements are presented. Independent on initial crystallinity, at oxygen concentrations > 2 at.% the Cu_2Zr_4O phase form primary to the main phases instead of simultaneously with the other main phases.

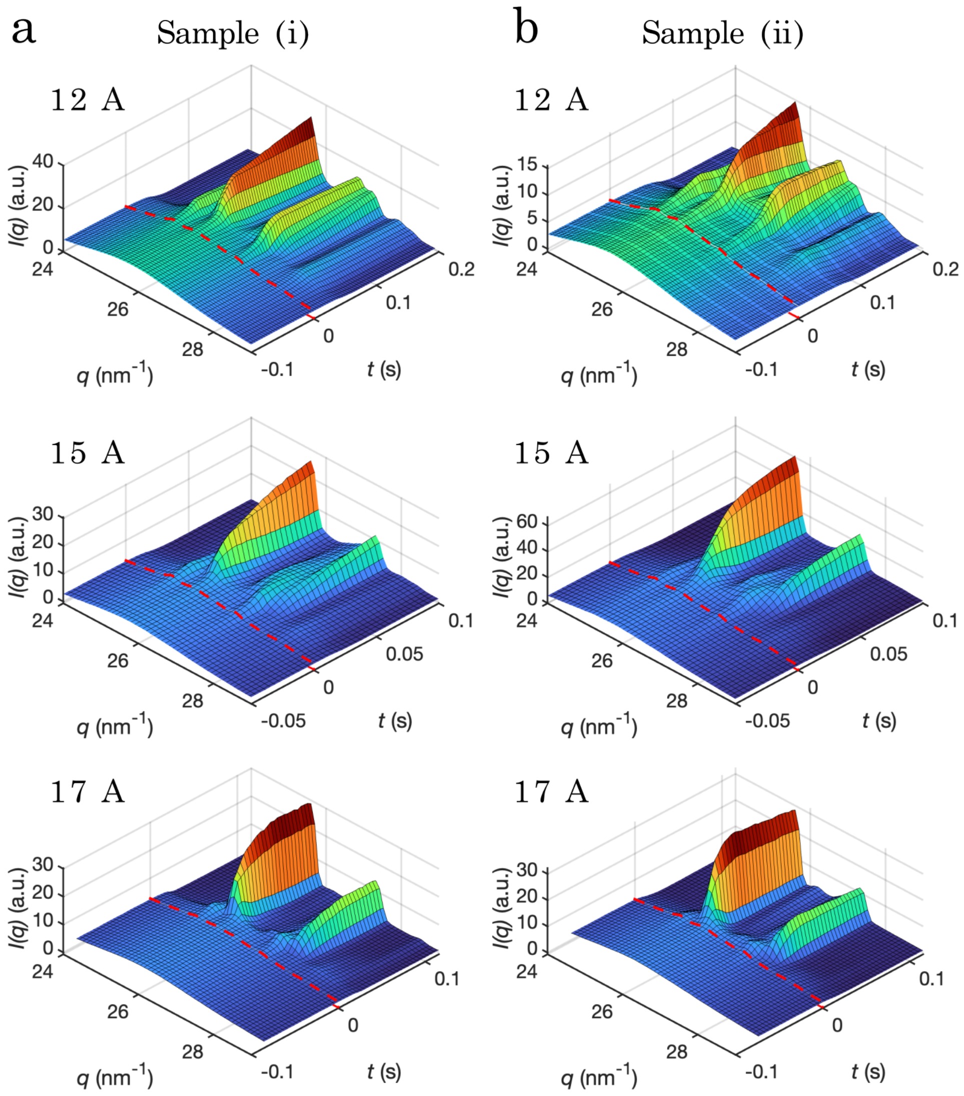

Figure S6: Structural ordering at onset of devitrification in samples (i) and (ii). **a** and **b**: WAXS patterns in the vicinity of t_{x1} for 12, 15, 17 A for sample (i) and (ii), respectively. Bragg reflections from all phases show simultaneously.

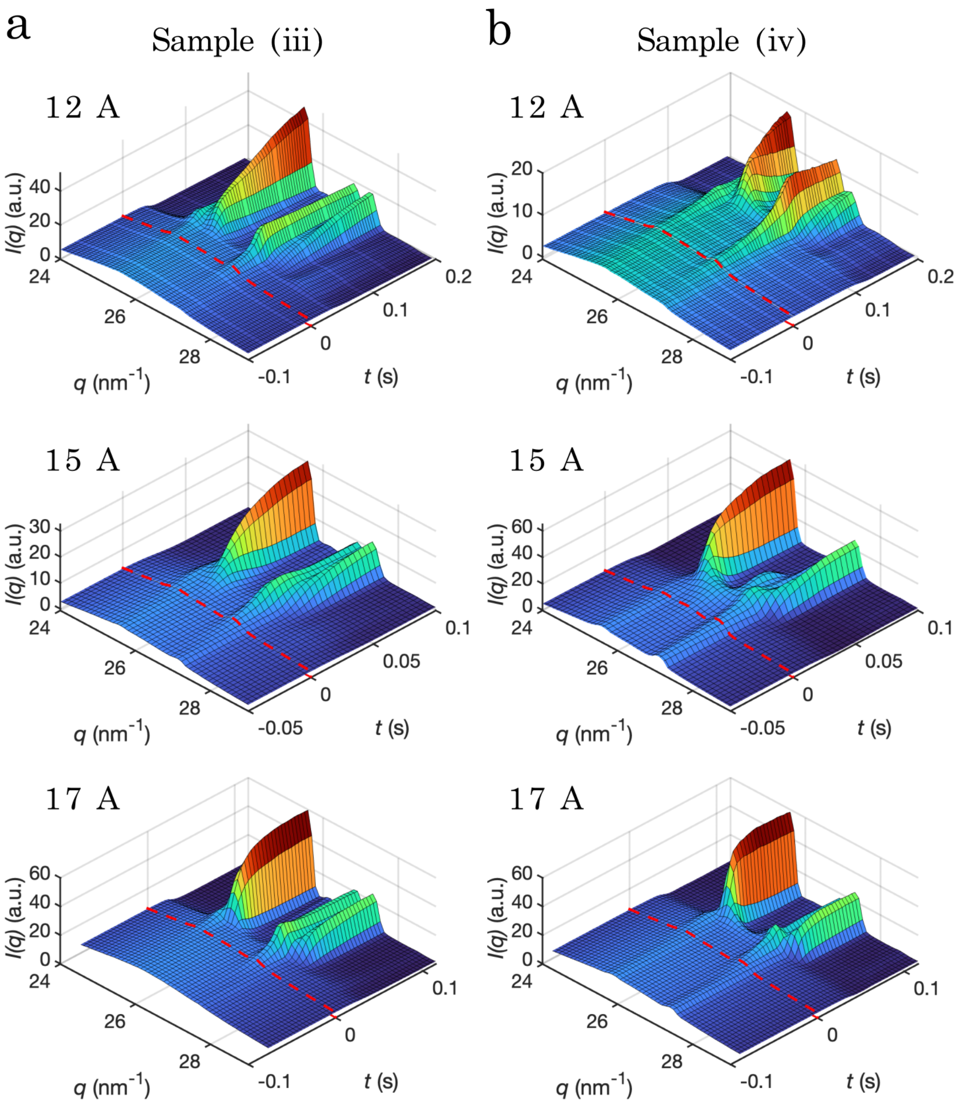

Figure S7: Structural ordering at onset of devitrification in samples (iii) and (iv). **a** and **b**: WAXS patterns in the vicinity of t_{x1} for 12, 15, 17 A for sample (iii) and (iv), respectively. Bragg reflections from the Cu_2Zr_4O phase show primary to the main phases, independent of initial crystallinity.

Microstructural analysis with TEM. In Figure S8, micrographs from the TEM analysis (bright field (BF), high angle annular dark filed (HAADF), selected area electron diffraction (SAED), and nano beam diffraction (NBD)) of samples (i) and (iv) are shown from which the $CuZr_2$, Al_3Zr_4, and Cu_2Zr_4O phases are identified. The identified lattice vectors have been indexed.

In Figure S9 the microstructure from HAADF STEM micrographs of sample (i) and (iv) flash-annealed with a current of 12 A are shown together with EDS micrographs. The dark areas, rich in Al, decrease in size with the increased oxygen content in sample (iv) compared to

sample (i) (Note the scale on the micrographs and compare to Figure S8d).

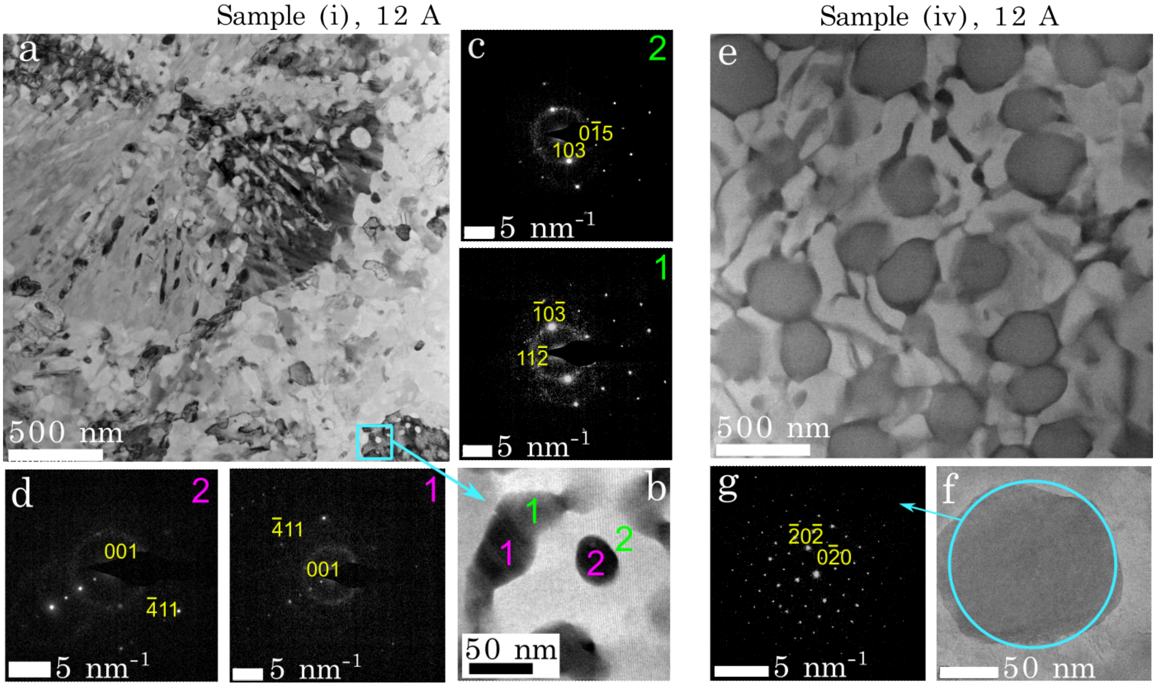

Figure S8: Phase identification with selected area electron diffraction and nano-beam diffraction. **a-d** Micrographs from TEM BF, STEM HAADF, and two sets of NBD from sample (i) subjected to flash-annealing from 12 A, respectively. In the bright and gray area of the STEM HAADF micrograph (**b**), green numbers mark the regions where the $CuZr_2$ phase could be identified and in the dark regions, magenta numbers mark the regions where the Al_3Zr_4 phase could be identified. **c** NBD with zone axis $\bar{3}51$ corresponding to the green numbers in **b**. **d** NBD with zone axis $\bar{1}\bar{4}0$ corresponding to the magenta numbers in **b**. **e-g** Micrographs from STEM HAADF, TEM BF, and SAED (Zone axis $\bar{1}01$) from sample (iv) subjected to flash-annealing from 12 A. Cu_2Zr_4O could be identified in the spherical/hexagonal particles seen in **e**.

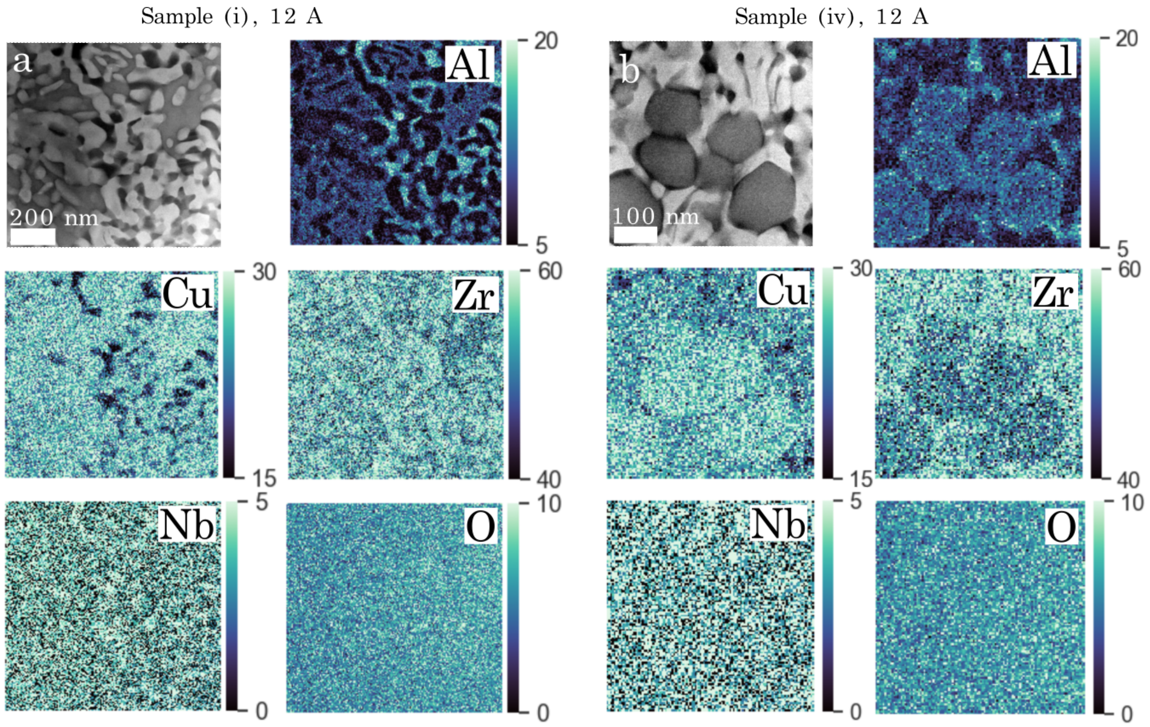

Figure S9: Microstructure of flash-annealed samples (i) and (iv) from a current of 12 A. **a** and **b**: STEM HAADF and EDS micrographs of the devitrified end product in samples (i) and (iv) from flash-annealing with a current of 12 A, respectively. The color bars represent relative atomic concentrations.

Parameter extraction from SAXS pattern. In Figure S10, the model fitting of selected SAXS patterns from flash-annealing of sample (iv) with a current of 12 A including the use of a structure factor, S, (S10a) and setting it to unity (S10b) are presented together with the extracted particle size distributions. In Figure S11a-c, the extracted distribution parameters from the model fitting of sample (i) and sample (iv), including the use of a structure factor and setting it to unity, subjected to flash-annealing with a current of 12 A are presented. In Figures S11a and b, the evolution of crystallized volume, V, number density, N, distribution width, σ_d, and the median precipitate radius, μ_d, are shown. The suggested transition between the two sets of results ((i)* and (iv)*) are marked with red and blue lines around dotted and dashed black lines, respectively. Fig S11c show the evolution of the local volume, η_d, and radius scaling, α_d, used in the structure factor.

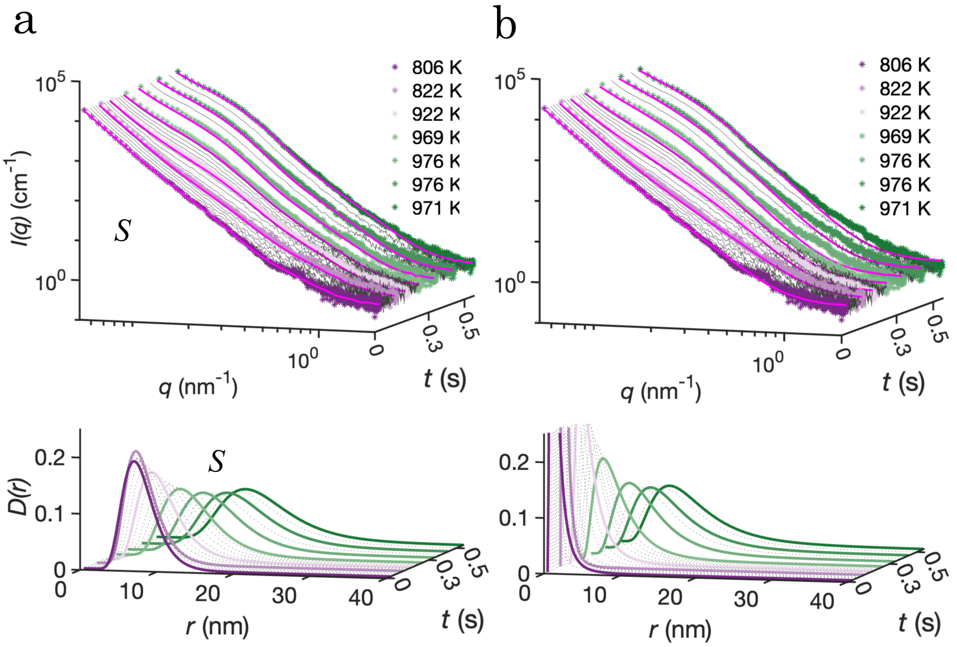

Figure S10: Model fitting of data from SAXS measurements during flash-annealing of sample (iv) with a current of 12 A. **a** and **b**: Selected SAXS patterns with model fitting in red together with computed PSD's using the structure factor, S, and setting it to unity, respectively.

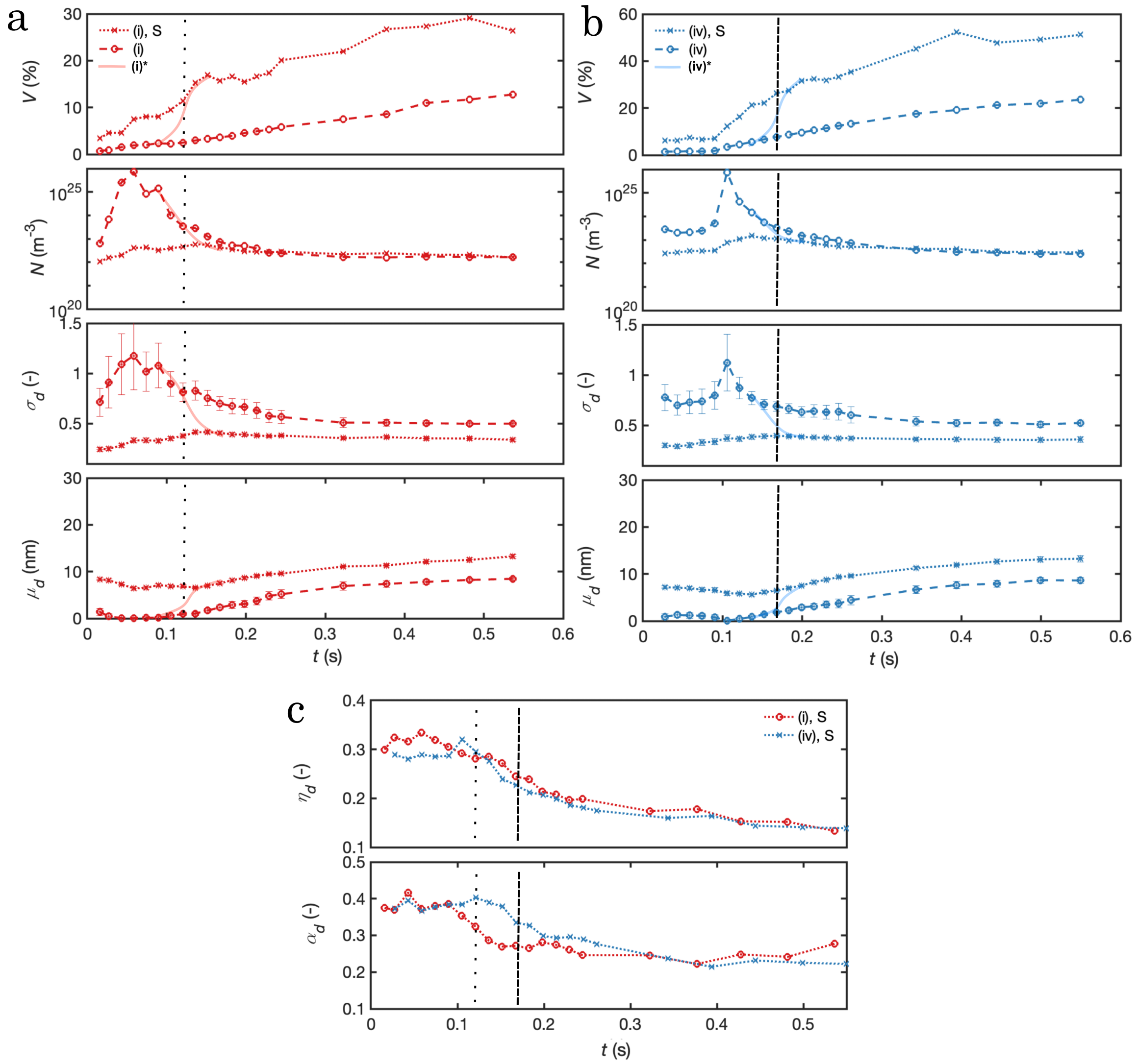

Figure S11: Extracted parameters from model fits of data from SAXS measurements during flash-annealing of samples (i) and (iv) with a current of 12 A. **a** and **b**: Parameters from resulting model fitting using the structure factor, S, (dotted lines), and setting it to unity (dashed lines) for sample (i) and (iv), respectively. From top; the volume fraction, V, number density, N, width of the distribution, σ_d, and the median radii, μ_d. A transition between the two different results ((i)* around a dotted black line and (iv)* around a dashed black line) are suggested based on the goodness of the fitting indicated by the reduced chi-square and the characteristic decrease in number density correlated to a more growth heavy transformation mode. **c** The local volume, η_d, and the radius scaling, α_d, which has been used as parameters when the structure factor has been applied.

Structural changes during melting and solidification in sample (iv) In Figure S12, WAXS analysis results from the flash-annealing of sample (iv) with a current of 17 A are presented. The temperature reached is high enough to melt the sample to a degree where the sample separate, this is seen as the spike in resistivity at $t = 1.7$ as well as the sharp drop in

intensity. However, the Cu_2Zr_4O phase is the dominant phase throughout the melting process and the first to show after the sample breakage.

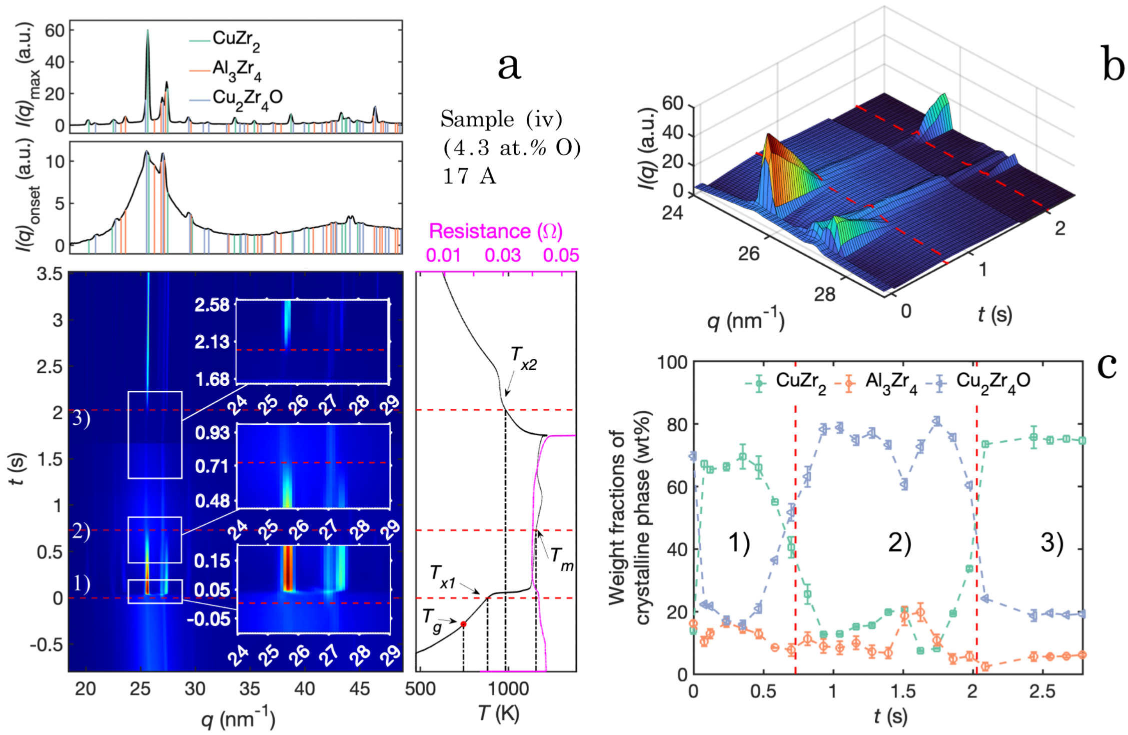

Figure S12: Structural phase ordering and disordering in sample (iv) containing 4.3 at.% O during flash-annealing with a current of 17 A resulting in an approximated linear heating rate $\Phi_{lin} = 740\,\mathrm{Ks}^{-1}$. Top **a**: Diffraction pattern at the maximum intensity during devitrification and at the onset of devitrification, t_{x1}. Bottom **a**: Integrated WAXS patterns from the flash-annealing measurements. It consists of three events: 1) devitrification when $0 < t < t_m$, 2) dissolution when $t_m < t < t_{x2}$ and, 3) crystallization when $t_{x2} < t$. Right bottom part of **a**: Time dependent temperature curves (black) and resistivity curves (pink). A sharp increase in temperature is observed at the exothermic nucleation event. The melting temperature T_m, the onset of crystallization T_{x2}, the onset of devitrification T_{x1}, and the glass transition temperature T_g, are marked with dashed black lines. The insets show close ups of the onset of devitrification, dissolution, and crystallization, respectively. **b** A 3D representation of the dissolution and crystallization events where the red dashed lines represent the onset of the events. **c** Weight phase fractions of the crystalline volume during the flash-annealing experiment starting from devitrification.
