# Supplementary material for: In Situ Mapping of Phase Evolutions in Rapidly Heated Zr‐Based Bulk Metallic Glass with Oxygen Impurities
Source: Adv Sci (Weinh). 2024 Feb 28;11(16):2307856. doi: 10.1002/advs.202307856 (PMC11040349; doi:10.1002/advs.202307856)
Supplement: Supplementary file 2 — Supporting Information [file ADVS-11-2307856-s002.pdf]

ADVANCED SCIENCE Open Access

Supporting Information

for *Adv. Sci.*, DOI 10.1002/advs.202307856

In Situ Mapping of Phase Evolutions in Rapidly Heated Zr-Based Bulk Metallic Glass with Oxygen Impurities

Mattias Tidefelt, Julia Löfstrand, Inga K. Goetz, Olivier Donzel-Gargand, Anders Ericsson, Xiaoliang Han, Petra E. Jönsson, Martin Sahlberg, Ivan Kaban and Martin Fisk*

In situ Mapping of Phase Evolutions in Rapidly Heated Zr-Based Bulk Metallic Glass with Oxygen Impurities

Mattias Tidefelt, Julia Löfstrand, Inga K. Goetz, Olivier Donzel-Gargand, Anders Ericsson, Xiaoliang Han, Petra E. Jönsson, Martin Sahlberg, Ivan Kaban, Martin Fisk*

Supporting Information: Classical nucleation and growth theory

The full description of the numerical model used in this work including a description of the energy barrier, thermodynamic considerations, nucleation and growth rate, and model implementation is presented below together with phase specific model parameters from the simulations.

Energy barrier for classical nucleation and growth theory. Considering a precipitating phase, β, with molar volume ,V_m^β, in a matrix phase, α, nucleation can be divided into two main categories, homogeneous and heterogeneous. For homogeneous nucleation, the change in Gibbs free energy as a function of cluster radius r can be expressed as[1]

$$\Delta G(r)_{hom} = -\frac{4\pi r^3}{3V_m^\beta} d_c + 4\pi r^2 \sigma_{\alpha\beta} \tag{1}$$

where d_c is the chemical driving force and $\sigma_{\alpha\beta}$ is the interfacial energy between the matrix phase and the precipitating phase. It describes the competing mechanism of volume-free energy release and the surface tension that arises during precipitation.

For heterogeneous nucleation, that results from the complete inclusion of a spherical particle belonging to phase γ with radius r_{core}, the Gibbs free energy can be expressed as[1]

$$\Delta G(r)_{het} = -\frac{4\pi(r^3 - r_{core}^3)}{3V_m^\beta} d_c + 4\pi(r^2 - r_{core}^2)\sigma_{\alpha\beta}. \tag{2}$$

A schematic illustration of the different approaches used to describe the formed clusters is shown in Figure S1.

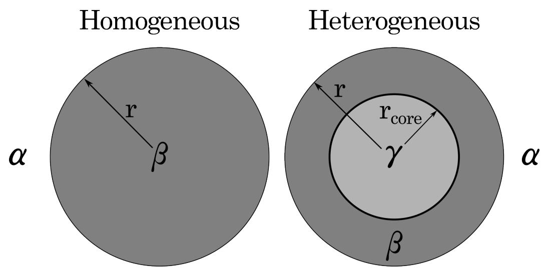

Figure S1: Different approaches used to describe the precipitating phase β in the matrix α with the Gibbs free-energy model. The homogeneous model is only described with a radii for the nuclei. To describe heterogeneous nucleation, two radii are used, one for the core, γ, and one for the shell of β phase.

The interfacial energy is very sensitive to small changes of nuclei size and, depending on how it is implemented, drastic changes in the simulated results can arise. Tolman developed a radius-dependent expression that accounts for the divergence from the sharp interface approximation at small cluster sizes[2], important for nuclei expected to form in BMG. By introducing a scaling variable Π to account for the inherent difficulties to describe the interfacial energy, the energy can be expressed as

$$\sigma_{\alpha\beta}(r) = \Pi \frac{\sigma_0}{1 + \frac{2\delta_0}{r}} \tag{3}$$

where σ_0 is the interfacial energy of a planar interface separating the two phases and δ_0 is a characteristic length scale. Turnbull[3] developed a theoretical expression for σ_0 considering the interfacial energy at the melting point with

$$\sigma_0 = \frac{\Delta H_m^f}{(V_m^\beta)^{2/3} N_A^{1/3}} \tag{4}$$

where N_A is Avogadro's constant and ΔH_m^f is the molar enthalpy of fusion. In this work ΔH_m^f is approximated by using tabulated data[1] for the elements in the stoichiometric composition, $A_m B_n$, such as

$$\Delta H_m^f = \frac{\mathrm{m}\Delta H_m^f(A) + \mathrm{n}\Delta H_m^f(B)}{\mathrm{m} + \mathrm{n}}. \tag{5}$$

The energy barrier for nucleation is found at the maximum of the energy function, i.e., $\partial \Delta G(r)/\partial r = 0$ and the solution gives the critical radius, r^*. For clusters of smaller radius it is energetically favorable to dissolve rather than to grow and vice versa. Inserting Eq. (3) in (1) gives the critical radius for homogeneous nucleation as

$$r^*_{hom} = -2\delta_0 + \frac{V_m^\beta \sigma_0}{d_c} + \sqrt{\frac{2V_m^\beta \sigma_0 \delta_0}{d_c} + \left(\frac{V_m^\beta \sigma_0}{d_c}\right)^2}. \tag{6}$$

Inserting Eq. (3) in (2) gives the critical radius for heterogeneous nucleation as the 4th order polynomial

$$r^*_{het} = -\frac{4\pi r^2 d_c}{V_m} + \frac{8\pi r^2 \sigma_0 (r + 3\delta_0 - \delta_0 (r_c/r)^2)}{(r + 2\delta_0)^2}. \tag{7}$$

The actual root is found by numerically solving the equation above with the starting guess taken as the well known expression[1] $r^*_{est} = 2\sigma^*_{est} V_m^\beta / d_c$ where $\sigma^*_{est} = \sigma_0 - \delta_0 d_c / V_m^\beta$ is used.

Thermodynamic considerations. Using the thermodynamic data base for the Al-Cu-Zr system developed by Zhou et al.[4], it is possible to model the precipitating phases in an approximation of AMZ4 matrix bulk phase as $Al_{10}Cu_{30}Zr_{60}$. With the molar fractions, x_i, of the constituent elements, the molar Gibbs free energy of the ternary bulk phase is described as a substitutional solution with[5]

$$G_m^\alpha = \sum_i^3 x_i G_i^\alpha + \mathrm{R}T \sum_i^3 x_i \ln(x_i) + {}^E G_m^\alpha, \quad \forall \quad i = \mathrm{Al, Cu, Zr} \tag{8}$$

where R is the universal gas constant and T is the temperature. Furthermore, G_i^α and ${}^E G_m^\alpha$ correspond to the reference energy of pure elements and the excess free energy. The excess term in the equation above takes into account all the non-ideal behavior of an alloy system and may be expressed using a Redlich-Kister expansion as

$${}^E G_m^\alpha = \sum_i^2 \sum_{j=i+1}^3 x_i x_j L_{ij}^\alpha + x_i x_j x_k L_{ijk}^\alpha \tag{9}$$

where L_{ij}^α and L_{ijk}^α correspond to the binary and ternary interaction parameters. The binary interaction parameters are temperature-dependent scalars and the ternary parameter is weighed as $L_{ijk}^\alpha = x_i L_{ijk}^{\alpha_i} + x_j L_{ijk}^{\alpha_j} + x_k L_{ijk}^{\alpha_k}$.[5]

The precipitating phases are described as line compounds modeled with sublattices as $(\mathrm{Al,Cu})_m\mathrm{Zr}_n$ where m and n represent the stoichiometric composition of the phase. Al and Cu are allowed to mix on the first sublattice, and Zr is held fixed on the second one. Thus, the elements Al and Cu are represented as site fractions, y', instead of molar fractions, and the Gibbs energy functions for the phases can be expressed as

$$G_m^\beta = \sum_i^2 y_i' G_{i:Zr}^\beta + m\mathrm{R}T \sum_i^2 y_i' \mathrm{ln} y_i' + y_{\mathrm{Al}}' y_{\mathrm{Cu}}' L_{\mathrm{Al,Cu,Zr}}^\beta. \tag{10}$$

The driving force is calculated using the maximum driving force approach from a common tangent construction between the matrix phase and the precipitating phase. For a ternary alloy system, the driving force can thus be expressed as[6]

$$d_c = \max\left(\sum_i^3 x_i^\beta \mu_i^\alpha(x_j^\alpha) - G_m^\beta(x_j^\beta)\right) \tag{11}$$

where x and μ represent molar fraction and the chemical driving force of component i at particle composition j.

Nucleation and growth rate. Constructed as an Arrhenius type relation with $\mathrm{k_B}$ as the Boltzmann constant and the energy barrier from either Eq. (1) or (2), the steady state rate of the number of nuclei formed can be expressed as[1]

$$J_{ss} = N_0 Z_f k^* \exp\left(-\frac{\Delta G(r^*)}{\mathrm{k_B}T}\right) \tag{12}$$

where N_0, Z_f and k^* corresponds to the number of nucleation sites, the Zeldovich factor and the condensation rate, respectively. Beside the above mentioned energy barrier, the number of nucleation sites is the other parameter that vary depending on if the nucleation event is considered to be homogeneous or heterogeneous. For homogeneous nucleation it is often approximated as $N_0^{hom} = N_A/V_m^\beta$ and for heterogeneous nucleation as $N_0^{het} = \Gamma N_0^{hom}$ where Γ is a fractional constant. The Zeldovich factor describes the probability for a nucleus to grow rather than dissolve at the critical size and can be expressed as[1]

$$Z = \frac{V_m^\beta}{2\pi r^{*2} N_A}\sqrt{\frac{\sigma(r^*)}{\mathrm{k_B}T}}. \tag{13}$$

The condensation rate describes how fast atoms attach to the growing clusters. Using an expression related to the atomic fractions of the system gives the condensation rate as[7]

$$k^* = \frac{4\pi r^{*2}}{\lambda^4} D_{\mathrm{eff}} \min(x_i^\alpha) \tag{14}$$

where D_{eff} is an effective diffusion coefficient and $\lambda = 2(3V_m^\alpha/4\pi N_A)^{1/3}$ is the atomic jump distance[1] with V_m^α as the molar volume of the matrix phase.

It is common to use the Einstein-Stokes relation when modeling diffusion in BMG[8,9] as it describes the diffusion of particles in a liquid. Assuming equal diffusivity of the constituent elements, the effective diffusion constant can be expressed as a function of viscosity, η, by[1]

$$D_{\text{eff}}(T) = \frac{\mathrm{k_B} T}{3\pi\lambda\eta} \tag{15}$$

However, because of the drastic change in viscosity as the liquid transforms to a supercooled and finally a glassy state, the expression fails to represent diffusion in BMG at large undercoolings. To represent the sluggish diffusion at higher viscosities better, Kelton et al.[10] developed a temperature-dependent Blodgett-Egami-Nussunow-Kelton (BENK) expression given by

$$\eta = \eta_0 \exp\left(\frac{E(T)}{T}\right) \tag{16}$$

where η_0 is the viscosity at infinite temperature which can be approximated as $\eta_0 = \mathrm{hN_A}/V_m^\alpha$ with h as Planck's constant, and $E(T)$ is an energy barrier. The barrier is expressed as

$$E(T) = E_\infty + T_A(aT_r)^b \Phi(T_A - T) \tag{17}$$

where T_A, E_∞ and T_r correspond to a universal scaling temperature approximated as $T_A = 2.02T_g$, a fitting parameter for the energy barrier approximated as $E_\infty = 6.466T_A$, and a reduced temperature. Further, $\Phi(X)$, a and b correspond to and a heavy side step function and universal fitting parameters.

As nucleation and growth in BMG depends on the supersaturation in the matrix, an expression that represent diffusion limited growth is needed. By assuming local equilibrium at the particle-matrix interface, an expression for the growth velocity is taken as[11]

$$v(x_i^\alpha - x_i^{\alpha\beta}) = \sum_{j=1}^{n-1} D_{ij}(x_j^{\beta\alpha} - x_j^{\alpha\beta})/\xi_j r \tag{18}$$

where $\xi = S/2K^2$. The parameter K is related to the super saturation

$$S = \frac{x_i^\alpha - x_i^{\alpha\beta}}{x_i^{\beta\alpha} - x_i^{\alpha\beta}}, \quad \forall \quad i = \mathrm{Al, Cu, Zr} \tag{19}$$

through

$$2K^2[1 - \sqrt{\pi}K \exp(K^2)\mathrm{erfc}(K)] = S. \tag{20}$$

The notation $\alpha\beta$ and $\beta\alpha$ represent the matrix and particle side of the interface respectively. For a ternary system, the growth rate as a function of r in Eq. 18 is solved with seven unknown variables. Keeping in mind that a system expressed with mole fractions has $n-1$ free elements, equilibrium equations can be constructed from[12]

$$\begin{aligned}
&\frac{\partial G_m^{\alpha,\beta}}{\partial x_i^{\alpha,\beta}} + \mu_i + \pi^{\alpha,\beta} = 0, \quad \text{all components in each phase} \\
&G_m^{\alpha,\beta} - \sum_{i=1}^{3} \mu_i x_i^{\alpha,\beta} = 0, \quad \text{all phases} \\
&\sum_{i=1}^{3} x_i^{\alpha,\beta} - 1 = 0, \quad \text{all phases}
\end{aligned} \tag{21}$$

where the variable $\pi^{\alpha,\beta}$ can be eliminated by substitution. The last condition needed is given by considering the Gibbs-Thomson effect as[9]

$$\mu_i^{\alpha}(x_j^{\alpha\beta}) = \mu_i^{\beta}(x_j^{\beta\alpha}) + \frac{2\sigma(r)V_m^{\beta}}{r}. \tag{22}$$

Model implementation. The structure of the implemented model is similar to that used in earlier work by Ericsson and Fisk[9], which was based on the model created by Myhr and Grong.[13] The crystallization is modeled with p phases. For each phase the time evolution of the number density distribution (NDD) is computed using an upwind scheme. With the time step, Δt, a heavy side step function, H, and the size class increment, Δr, it can be computed as[14]

$$\begin{aligned}
N_{i,t+\Delta t} = N_{i,t} + \frac{\Delta t}{\Delta r}&(v_{i-1,t}(H(v_{i-1,t})N_{i-1,t} + H(-v_{i-1,t})N_{i,t}) \\
&-v_{i,t}(H(v_{i,t})N_{i,t} + H(-v_{i,t})N_{i+1,t})).
\end{aligned} \tag{23}$$

To keep the distribution stable during the simulations, the time step needs to be restricted so that the clusters do not grow through multiple cluster size bins at once. This is done by choosing the time step as

$$\Delta t = \min\left(\frac{\gamma}{2}\min\left(\frac{\Delta r_\beta}{|v_\beta|}\right)\Bigg|_{\beta=1:p}\right). \tag{24}$$

Here γ is a factor taken such that the solution is stable, in this work it is taken as 0.9. Keeping mass balance, each phase computed crystalline volume fraction contributes to the change in matrix composition as

$$x_m(1 - f_{tot}) = x_0 - \sum_{i=1}^{p} f_i x_i \tag{25}$$

where x_0, x_m and $f_{tot} = \sum_i^p f_i$ represent the initial matrix composition, the varying matrix composition and the total fraction of the crystalline volume, respectively. As the particles are approximated as spheres, the volume fraction is computed as $f = 4\pi/3 \sum_i N_i r_i^3$. This methodology can easily be extended to incorporate more phases as the phase contributions are computed by iterating the same sequence as for single-phase modeling.

Modeling results. In the Figure S2a and S2b, the computed steady-state nucleation rate, J_{ss}, and maximum of the growth rate, v, as a function of temperature for homogeneous and heterogeneous nucleation modes are presented, respectively. Here we see how the introduced heterogeneous nucleation mode impact the nucleation rate, shifting the maximum towards higher temperatures for both phases, but increase the rate more for the $CuZr_2$ phase more than for the Al_3Zr_4 phase.

In Figure S3, the composition of the particles as a function of radius, extracted from the growth function under the local equilibrium condition, are presented for the homogeneous and heterogeneous nucleation modes. For clusters with a radii smaller than 10 nm, small variations are observed. At larger radii, the composition in the particles become constant.

In Figure S4a and S4b, the computed growth parameters of the $CuZr_2$ and Al_3Zr_4 phases at the end of the simulations, $t_{30\%}$, as a function of particle radius for homogeneous and heterogeneous nucleation modes are presented, respectively. The growth rate, and supersaturation, S, and composition at the matrix side of the particle-matrix interface are all affected by the heterogeneous nucleation mode. The $CuZr_2$ is affected the most where a twice as strong growth rate is observed whereas the Al_3Zr_4 phase show a reduced supersaturation when the heterogeneous mode is applied.

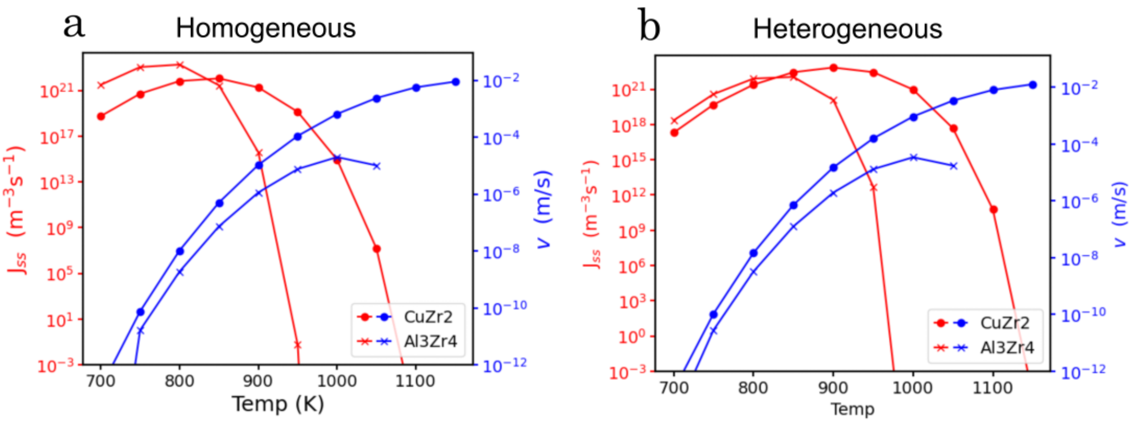

Figure S2: Temperature dependent devitrification model parameters. **a** and **b** Maximum growth rate and nucleation rate as a function of temperature for homogeneous and heterogeneous nucleation modes, respectively.

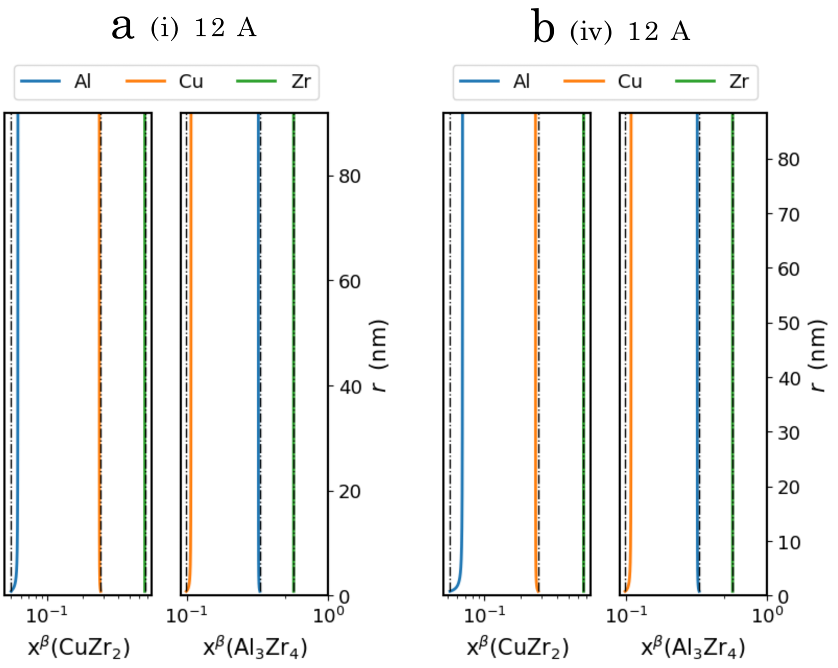

Figure S3: Radius dependent particle composition extracted at the end of the simulations. **a** and **b** correspond to homogeneous and heterogeneous nucleation modes, respectively, simulated with the temperature data from samples (i) and (iv) subjected to flash annealing from 12 A.

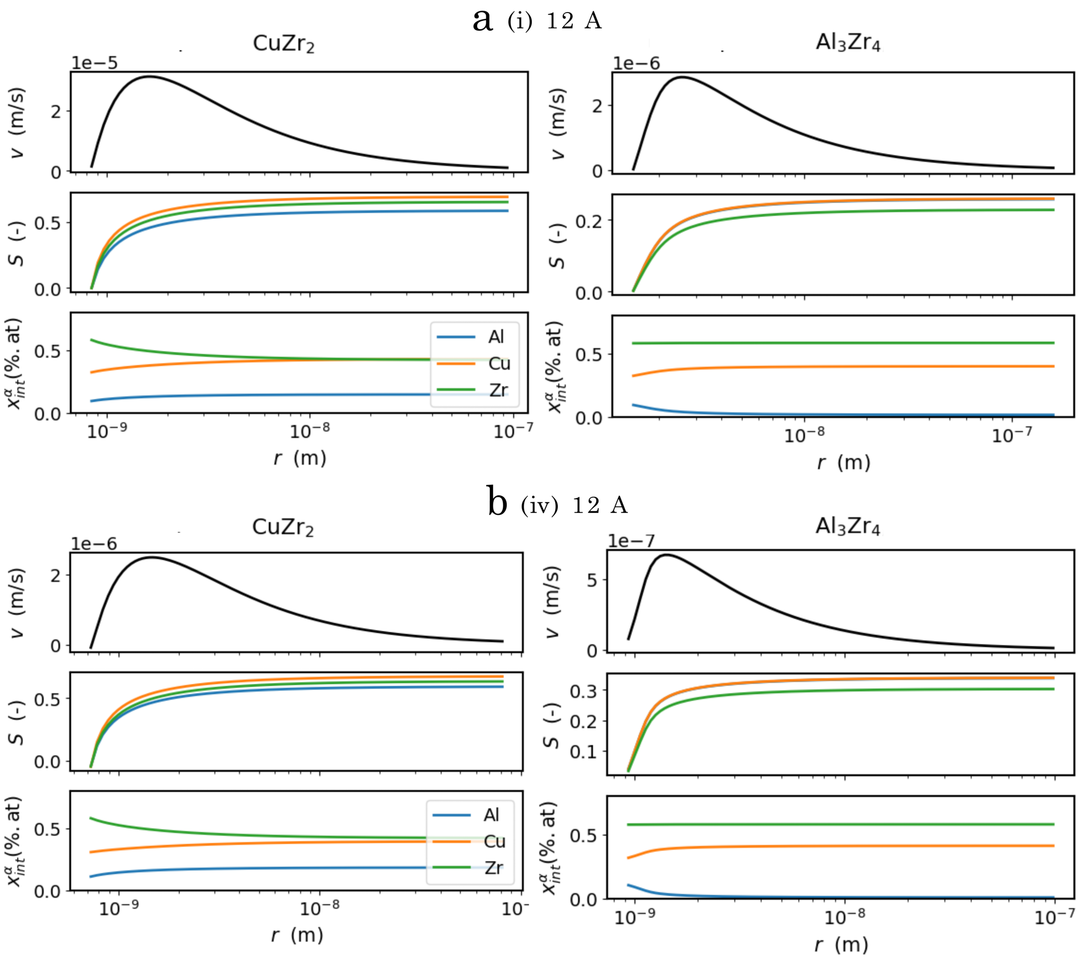

Figure S4: Growth parameters for the two different phases at $t_{30\%}$. **a** and **b** Growth velocity, element specific supersaturation, and particle composition at the matrix side of the interface from simulations using the temperature data from samples (i) and (iv) subjected to flash annealing from 12 A.

References

[1] K. F. Kelton, A. L. Greer, *Nucleation in Condensed Matter: Applications in Materials and Biology*, Pergamon, Elsevier Ltd, Linacre House, Jordan Hill, Oxford OX2 8DP, UK, **2010**.

[2] R. C. Tolman, *The Journal of Chemical Physics* **1949**, *17*, 333. `https://doi.org/10.1063/1.1747247`.

[3] D. Turnbull, *The Journal of Chemical Physics* **1950**, *18*, 769. `https://doi.org/10.1063/1.1747766`.

[4] C. Zhou, C. Guo, C. Li, Z. Du, *J. Non-Cryst. Solids* **2017**, *461*, 47. https://doi.org/10.1016/j.jnoncrysol.2016.09.031.

[5] M. Hillert, *Phase Equilibria, Phase Diagrams and Phase Transformations*, Cambridge University Press, Cambridge CB2 8RU, UK, **2008**.

[6] B. Rheingans, E. J. Mittemeijer, *Calphad: Computer Coupling of Phase Diagrams and Thermochemistry* **2015**, *50*, 49. https://doi.org/10.1016/j.calphad.2015.04.013.

[7] T. Philippe, M. Bonvalet, D. Blavette, *Journal of Chemical Physics* **2016**, *144*, 20. https://doi.org/10.1063/1.4950878.

[8] Z. Yang, R. Al-Mukadam, M. Stolpe, M. Markl, J. Deubener, C. Körner, *J. Non-Cryst. Solids* **2021**, *573*, 121145. https://doi.org/10.1016/j.jnoncrysol.2021.121145.

[9] A. Ericsson, M. Fisk, *Metals* **2022**, *12*, 867. https://doi.org/10.3390/met12050867.

[10] M. E. Blodgett, T. Egami, Z. Nussinov, K. F. Kelton, *Sci. Rep.* **2015**, *5*, 1. https://doi.org/10.1038/srep13837.

[11] Q. Chen, J. Jeppsson, J. Ågren, *Acta Mater.* **2008**, *56*, 1890. https://doi.org/10.1016/j.actamat.2007.12.037.

[12] H. L. Lukas, S. G. Fries, B. Sundman, *Computational thermodynamics: The Calphad method*, Cambridge University Press, **2007**.

[13] Myhr O.R., Grong Ø., *Acta Mater.* **2000**, *48*, 1605. https://doi.org/10.1016/S1359-6454(99)00435-8.

[14] M. Perez, M. Dumont, D. Acevedo-Reyes, *Acta Mater.* **2008**, *56*, 2119. https://doi.org/10.1016/j.actamat.2007.12.050.
